# Dissecting residual disease in spheroids reveals pan-cancer persistence signatures and a therapeutic window for oncolytic viruses

**DOI:** 10.1016/j.omton.2026.201279

**Published:** 2026-06-19

**Authors:** Clara Fauveau, Emily Lawendy, Jules Deforges, Sandrine Cochin, Baptiste Moreau, Jean-Marc Balloul, Philippe Erbs, Shreyansh Jain, Gilles Laverny, PERSIST-SEQ Consortium

**Affiliations:** 1 Transgene, 67400 Illkirch-Graffenstaden, France; 2 Institute of Genetics and Molecular and Cellular Biology (IGBMC), 67404 Illkirch-Graffenstaden, France; 3 CNRS UMR 7104, 67404 Illkirch-Graffenstaden, France; 4 Inserm U1258, 67404 Illkirch-Graffenstaden, France; 5 University of Strasbourg, 67404 Illkirch-Graffenstaden, France

**Keywords:** oncology, persistence, non-small cell lung cancer, chemotherapy, spheroid, single-cell RNA-seq, oncolytic viruses

## Abstract

Drug resistance remains a major burden in clinical care, often emerging from a subpopulation of cells in a drug-tolerant state. In this study, we aimed to characterize the transcriptional features of persistent non-small cell lung cancer (NSCLC) cells following cisplatin-pemetrexed chemotherapy and explore the therapeutic potency of oncolytic viruses to eliminate these cells. We established a 3D spheroid model of NSCLC and applied long-term chemotherapy to induce a reversible, non-proliferative persistent state associated with lower sensitivity to treatment. Single-cell RNA sequencing coupled with comparative analysis of multiple human datasets sheds light on a core transcriptional signature of persistence. This signature was enriched in patient-derived minimal residual disease (MRD) datasets, highlighting the clinical relevance of persistent preclinical models. Furthermore, transcriptomic analyses suggested a vulnerability of persister cells to oncolytic viruses, a finding validated in spheroid and patient-derived organoids. Altogether, these results define a conserved persistence signature and support the use of virotherapy as a promising option to target MRD.

## Graphical abstract

**Figure:**
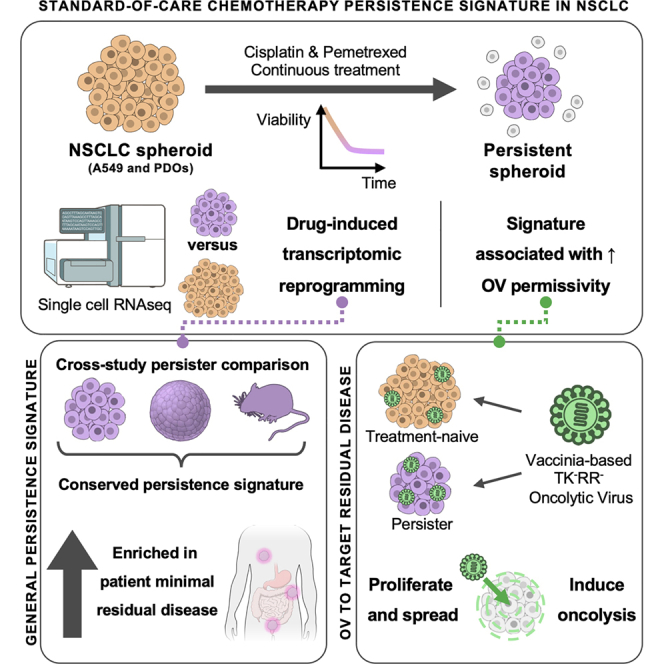

## Teaser

In this study, Laverny and colleagues report a conserved persistence signature shared across preclinical models and patient minimal residual diseases and identify oncolytic viruses as a candidate therapeutic strategy to eliminate persister cells.

## Introduction

Treatment resistance remains a major burden in the care of almost all cancer types. Among the 9.6 million deaths per year due to cancer, 90% are attributable to an untreatable relapse following an initial effective treatment.^1^ A critical contributor to this failure is the survival of a small population of tumor cells during therapy, known as minimal residual disease (MRD), that serves as a quiescent reservoir for relapse.^2^^,^^3^ While MRD often harbors pre-existing resistant clones, several studies have also identified residual tumor cells with minimal evidence of clonal selection, pointing to the persistence of a non-genetically encoded drug-tolerant state.^4^^,^^5^^,^^6^

Drug-tolerant persister cells (DTPs) represent a reversible, adaptive, non-genetic state induced by therapeutic pressures, observed across multiple tumor types, both *in vitro* and *in vivo*. These cells undergo reversible phenotypic changes including cell-cycle arrest, drug cross-tolerance, and epigenetic reprogramming.^7^ The transcriptomic profile of DTPs closely resembles the one observed in MRD patients, supporting the hypothesis that DTPs constitute a major component of MRD and serve as a reservoir, from which relapse eventually arises.^8^ Given their pivotal role in treatment failure, there is a growing focus on characterizing and targeting DTPs before the emergence of irreversible resistance mechanisms.^9^

Non-small cell lung cancer (NSCLC) affects over 2 million individuals worldwide each year. Despite advances in targeted therapies such as epidermal growth factor receptor (EGFR) tyrosine kinase inhibitors (TKIs), immune checkpoint inhibitors, and platinum-based chemotherapy, the long-term outcome remains poor, with only 25% of the patients surviving beyond 5 years after diagnosis.^10^ While several studies have explored MRD signatures upon EGFR TKI treatment,^7^^,^^11^^,^^12^^,^^13^ the drug-tolerant state arising after cisplatin and pemetrexed, the standard-of-care chemotherapy, remains to be characterized.

One innovative approach to cancer therapy involves the use of oncolytic viruses (OVs), which are genetically modified to selectively infect and lyse cancer cells.^14^ Beyond oncolysis, engineered OVs carrying therapeutic transgenes within their viral backbones were developed, enabling local delivery of immunomodulatory or cytotoxic agents.^15^^,^^16^ Although OVs have shown promise in treating solid tumors,^17^^,^^18^^,^^19^ their effectiveness in a persistent context remains largely unexplored. Several *in vitro* studies have yielded conflicting results regarding the efficacy of OV-based treatments following chemotherapy exposure,^20^^,^^21^^,^^22^ highlighting the need for further research to determine OV efficacy in a chemotherapy-induced persistent context.

To address this gap, we aimed to establish a preclinical model that mimics the emergence of DTPs following chemotherapy. We used the NSCLC-derived A549 cell line, which has been extensively characterized for its response to chemotherapy and targeted agents.^23^ Cells were cultured as three-dimensional (3D) spheroids. Compared with 2D cultures, 3D spheroids better recapitulate tumor heterogeneity, cell-extracellular matrix interactions,^24^ and hypoxic gradients,^25^ thereby providing clinically relevant drug responses.^26^^,^^27^^,^^28^^,^^29^ This study models an NSCLC MRD-like persistent state by treating A549 spheroids with a clinically relevant cisplatin-pemetrexed regimen and uses single-cell transcriptomic analysis to define the resulting persistence signature. In addition, data mining of multiple DTP preclinical studies identified conserved hallmarks of the persistent state that are enriched in MRD patient datasets. Finally, the current study evaluates the efficacy of a vaccinia virus (VACV)-based OV, double-deleted for the thymidine kinase (TK) and ribonucleotide reductase (RR),^30^ in targeting DTPs generated from both NSCLC spheroids and patient-derived organoids (PDOs).

## Results

### Emergence of drug-tolerant cells under NSCLC standard-of-care chemotherapy

**Figure 1:**
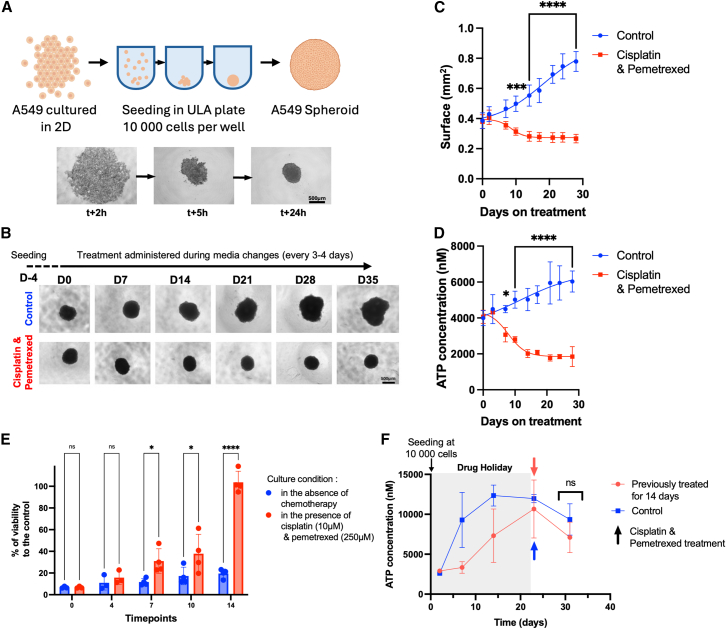
Modeling chemotherapy persistence in 3D spheroids (A) Schematic representation of A549 spheroid formation using Ultra-Low Attachment (ULA) plates, alongside images showing the aggregation 2, 5, and 24 h post seeding. Scale bar, 500 μm. (B) Representative bright-field images of A549 spheroids in the presence of 10 μM cisplatin and 250 μM pemetrexed, or vehicle over time. Scale bar, 500 μm. (C) Surface area evolution of A549 spheroids treated with 10 μM cisplatin and 250 μM pemetrexed, or with vehicle for the indicated time. Thirty-six spheroids were analyzed per condition/time point from three biological replicates; mean (SD); ∗∗∗*p* < 0.001 and ∗∗∗∗*p* < 0.0001 vs. control spheroids, determined using two-way ANOVA followed by a Šídák’s multiple comparisons test. (D) Longitudinal ATP content in A549 spheroids treated with 10 μM cisplatin and 250 μM pemetrexed, or with vehicle for the indicated time. Nine spheroids per condition/time point from three biological replicates; mean (SD); ∗*p* < 0.05 and ∗∗∗∗*p* < 0.0001 vs. control spheroids, determined using two-way ANOVA followed by a Šídák’s multiple comparisons test. (E) Spheroids were cultured in the presence or absence of chemotherapy (10 μM cisplatin and 250 μM pemetrexed) for the indicated time points and then the viability was determined upon a challenge with a high-dose bolus of chemotherapy (100 μM cisplatin and 2,500 μM pemetrexed) or vehicle for 5 days. Data are expressed as the viability relative to the spheroids challenged with vehicle. Nine spheroids per condition/time point from three biological replicates; mean (SD); ns, *p* ≥ 0.05; ∗*p* < 0.05 and ∗∗∗∗*p* < 0.0001 vs. matched spheroids challenged with vehicle, determined using two-way ANOVA followed by a Šídák’s multiple comparisons test. (F) Spheroids were treated for 14 days with the chemotherapy or vehicle. Once dissociated and seeded, the ATP content was monitored for 24 days in drug-free conditions. At day 24, treatment-naive (blue curve) and post-drug holiday (red curve) spheroids were treated for 8 days with 10 μM cisplatin and 250 μM pemetrexed (represented by arrows) and the ATP content was determined. Nine spheroids per condition/time point, in three biological replicates; mean (SD); ns *p* ≥ 0.05 vs. control spheroids, determined using an unpaired *t* test.

We first set up a reproducible spheroid model using the immortalized A549 cell line derived from NSCLC. Over a 24-h period, cells seeded into an ultra-low attachment plate aggregated, resulting in the formation of a single 3D A549 spheroid per well (Figure 1A). Over a month of culture, these spheroids grew to 0.8 mm^2^, developing heterogeneous shapes and uneven edges (Figures 1B and 1C). The ATP content from lysed individual spheroids, determined by CellTiter-Glo assay, increased in a time-dependent manner (Figure 1D), demonstrating their continuous growth over the period of culture. This stable and proliferative baseline condition supports the use of spheroids for evaluating the impact of chemotherapy.

To determine the effect of cycles of standard-of-care chemotherapy, spheroids were exposed every 3–4 days to a combination of 250 μM pemetrexed and 10 μM cisplatin, concentrations reflecting peak plasma levels observed in patients following intravenous administration.^31^ The size of the chemotherapy-treated spheroids decreased while remaining smooth edged and round shaped, in contrast to the vehicle-treated ones (control) (Figure 1B). Spheroid size and ATP content decreased over the first 10 days of treatment and then reached a plateau (Figures 1C and 1D). In line with these results, treated spheroids became progressively less sensitive to chemotherapy, with 90% viability following a high-dose bolus, compared with only 19% in spheroids not pre-exposed to chemotherapy for 14 days (Figure 1E). Taken together, these results showed that the treatment impacts spheroids with a biphasic dynamic, featuring an initial phase characterized by a reduction of the cell population, followed by a second one associated with a stalled growth and a reduced sensitivity to treatment.

We then compared the growth of treated and control spheroids cultured in a drug-free media. Spheroids treated for 14 days and matched-time point controls were dissociated and seeded with the same number of cells. While the ATP content of spheroids derived from control cells increased from the first day of culture, those obtained from treated cells resumed proliferation with an 8-days delay (Figure 1F). Strikingly, they both reached similar levels around 22 days of culture. Post-drug holiday, spheroids also regained a treatment sensitivity comparable to untreated controls, indicating that both the proliferation arrest and the decrease in sensitivity observed previously were reversible. Thus, the characteristics of the treated 3D spheroids align with the features of DTPs.^32^

### Single-cell transcriptomic signature of persistent spheroids

**Figure 2:**
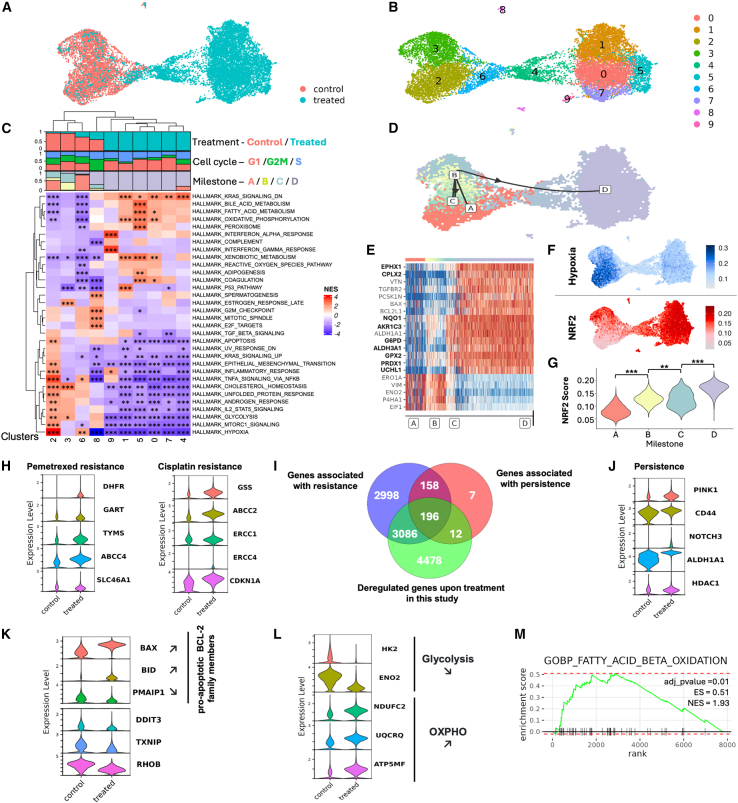
Trajectory and signatures of control and chemotherapy-treated A549 spheroids UMAP representation of cells colored by treatment conditions (control vs. treated) (A) and by unsupervised clusters (B). (C) Heatmap showing the normalized enrichment scores (NESs) of the Hallmarks across clusters. Top bar plots indicate the percent of cells belonging to the treatment condition, cell cycle phase, or the trajectory milestone (D) per cluster. The stars depict the significance of the adjusted *p* value. ∗*p* < 0.05, ∗∗*p* < 0.01, and ∗∗∗*p* < 0.001 vs. the other clusters, determined using an adaptive multi-level split Monte-Carlo method. (D) Trajectory analysis where the line and the arrow indicate the inferred lineage trajectory. The colors represent the cells belonging to the various milestones (A–D). (E) Heatmap representing the important genes characterizing the trajectory. Those in bold are associated with the NRF2 signature.^33^ (F) UMAP representation of cells colored by the enrichment score for the Hallmark Hypoxia signature (top) and the NRF2 signature (bottom). (G) Violin plot of the enrichment score for the NRF2 signature across trajectory milestones. ∗∗*p* < 0.01 and ∗∗∗*p* < 0.001 vs. the next milestone, determined using pairwise Wilcoxon tests. (H) Violin plots representing the transcript levels of significantly deregulated genes associated with reduced sensitivity to pemetrexed or cisplatin. (I) Venn diagram representing the number of deregulated genes identified in our study and genes associated with resistance or persistence in the literature. Violin plots representing the transcript levels of significantly deregulated genes associated with persistence (J), apoptosis (K), and glycolysis and oxidative phosphorylation pathways (L). (M) Enrichment plot of the fatty acid β-oxidation biological process upon treatment. Adjusted *p* value computed using an adaptive multi-level split Monte-Carlo method.

To uncover the transcriptional changes associated with sustained chemotherapy exposure, 14,262 cells from control spheroids and from spheroids treated for 14 days were analyzed in multiple biological replicates by droplet-based single-cell RNA sequencing (scRNA-seq) (Figures S1A, S1B, and S1C). Uniform manifold approximation and projection (UMAP) visualization revealed that the cells clustered based on their treatment, with a marginal bridging between the two conditions (Figure 2A). The unsupervised clustering revealed 10 distinct groups of cells (Figures 2B and S1D; Table S1). Cell-cycle phase distribution and Molecular Signatures DataBase (MsigDB) hallmark enrichment were assessed for each cluster (Figure 2C). Clusters 2, 3, 6, and 8 were mainly composed of control cells. Apart from cluster 8, which regrouped cells in S and G2M phases and associated with proliferative hallmarks, all the other control clusters showed a balanced proportion of cells in the different cell cycle phases (Figure 2C). Cluster 2 exhibited a hypoxic signature, elevated inflammatory and epithelial-mesenchymal transition (EMT) signatures, as well as a metabolic profile dominated by glycolysis. Cluster 6 was characterized by an absence of glycolysis enrichment and a milder hypoxic profile, whereas cluster 3 displayed no significant deregulation in inflammation, oxidative phosphorylation, or hypoxia compared with the other control clusters. In contrast to the heterogeneity observed in control cells, clusters of the treated cells presented uniform enrichments, with consistent downregulation of multiple pathways and a 31% reduction in the proportion of cells in the G2/M phase.

To gain deeper insight into the cellular heterogeneity and plasticity, we performed a cell trajectory analysis that uncovered a linear progression structured into four different milestones (Figures 2C and 2D; Table S2). Within the control condition, cells were distributed across three distinct states. Cells in milestone A (mainly clusters 2 and 6) were characterized by markers of anaerobic glycolysis (e.g., *ENO2*) and an EMT phenotype (e.g., *VIM* and *P4HA1*) (Figure 2E), while cells in milestone C (clusters 2 and 3) displayed activation of the transcription factor NRF2, a master regulator of cellular antioxidant response with upregulation of several target genes (e.g., *NQO1*, *ALDH1A1*, *GPX2*, and *PRDX1*). Milestone B represented an intermediate state between milestones A and C (Figure 2E). Within control cells, NRF2 expression was inversely correlated to the hypoxia hallmark signature (Figure 2F), consistent with reactive oxygen species formation and NRF2 activation being dependent on oxygen availability. The presence of an oxygen gradient within control spheroids, ∼800 μm in diameter at the time of the scRNA-seq, is supported by the continuous increase of hypoxia-related gene expression along control spheroid growth (Figure S2A).

In contrast, the treated condition was largely dominated by a single trajectory state, the milestone D, characterized by an overall enrichment of the NRF2 signature (Figures 2E, 2G, and S2B). Quantification of the expression of five NRF2 target genes further confirmed pathway activation over the course of the treatment (Figure S2C).

Overall, the trajectory captures a continuum of oxidative stress adaptation, progressing from hypoxic cells with low NRF2 activity, to more oxygenated cells with increasing NRF2 activation, and finally to treated cells in which this program is fully activated.

Pseudo-bulk differential gene expression analysis revealed that, among the 18,082 genes detected, a total of 4,764 genes were significantly upregulated and 3,008 were downregulated when comparing treated versus control conditions (Tables S3 and S4). Genes encoding pemetrexed’s direct targets (DHFR, GART, and TYMS^34^^,^^35^); ABCC4, a pemetrexed exporter; or SLC46A1, involved in folate uptake,^36^ were all upregulated (Figure 2H). Upregulation was also observed for genes encoding proteins involved in cisplatin inactivation and export such as GSS or ABCC2, and proteins involved in DNA repair (e.g., ERCC1,^37^ ERCC4,^38^^,^^39^ and CDKN1A^40^) (Figure 2H). Mining in PubMed abstracts identified 6,483 genes linked to treatment resistance, including 43% also deregulated in our model (Figure 2I; Table S5). Applying the same approach to persistence revealed an even higher overlap with 61% of the identified genes deregulated in our model (Figure 2I; Table S5). Among these genes were those encoding PINK1, known for its role in mitophagy^41^; stem cell markers CD44, NOTCH3, and ALDH1A1^42^^,^^43^; as well as HDAC1 involved in epigenetic deregulation in DTPs^7^^,^^44^ (Figure 2J).

Cells’ effort to cope with the treatment was visible via an increase of the xenobiotic metabolism pathway (Figure 2C). The p53 pathway, and several pro-apoptotic members of the BCL-2 family, were significantly deregulated in opposing directions (Figure 2K). However, genes involved in apoptosis regulation, such as *DDIT3*, *TXNIP*, or *RHOB*, were consistently downregulated (Figure 2K), as was the apoptosis pathway itself (Figure 2C), thereby supporting the survival of persister cells. The most striking change upon treatment involved metabolic rewiring, with a switch from glycolysis (with a downregulation of *HK2* and *ENO2* for instance) to oxidative phosphorylation (OXPHOS), characterized by an upregulation of genes such as *NDUFC2*, *UQCRQ*, and *ATP5MF* (Figure 2L), all components of the mitochondrial electron transport chain. Moreover, fatty acid metabolism, and particularly β-oxidation, which generates NADPH, were also upregulated (Figures 2M and S3A). These shifts have been previously described as key metabolic changes promoting DTP survival.^11^^,^^45^

Finally, treated cells exhibited a reduced expression of EMT-related genes compared with controls (Figure 2C). Note that despite *CD44*, *NOTCH3*, or *ALDH1A1* upregulation, signatures of cancer stem cells or senescent cells were not enriched in treated cells (Figures S3B and S3C).

Together, these results indicate that chemotherapy-persistent A549 spheroids are characterized by (1) a reduced proportion of cells in G2/M and proliferation; (2) upregulation of genes associated with cisplatin and pemetrexed low response; (3) broad metabolic reprogramming, including decreased glycolysis, increased oxidative phosphorylation, and fatty acid oxidation; and (4) activation of the NRF2 pathway, all hallmarks of DTPs.

### Conserved changes upon persistence in preclinical models and in patients

**Figure 3:**
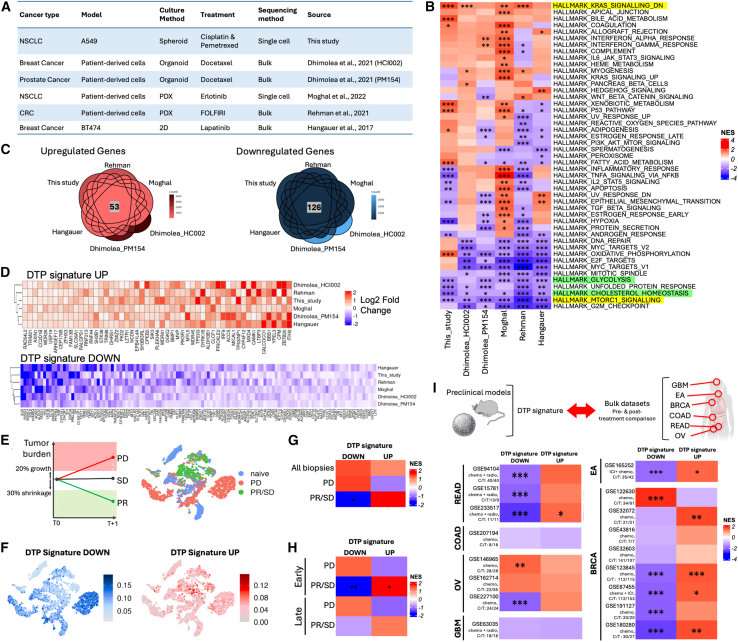
Multi-model comparison of drug-tolerant persisters (A) Summary of the DTP transcriptome datasets used for the comparative analysis. (B) Heatmap of deregulated Hallmarks following persistence induction across datasets. The color scale represents the normalized enrichment score (NES), and the stars indicate the significance of the adjusted *p* value. Conserved signaling hallmarks are highlighted in yellow, while processes under their regulation are shown in green. The stars depict the significance of the adjusted *p* value. ∗*p* < 0.05, ∗∗*p* < 0.01, and ∗∗∗*p* < 0.001 vs. control cells, determined using an adaptive multi-level split Monte-Carlo method. (C) Venn diagram of significantly deregulated genes in the DTP datasets. The red diagram shows the upregulated genes, while the blue one shows those downregulated. (D) Heatmaps representing the fold change of the core DTP signature genes (53 up- and 126 down-regulated, C) in each DTP dataset. (E) UMAP representation of NSCLC patient’s epithelial cells^12^ colored by the disease status (naive, untreated; PD, progressive disease; SD, stable disease; PR, partial response) at the time of biopsy. (F) UMAP plots of NSCLC patient’s epithelial cells^12^ colored by the core DTP signature scores. (G) Heatmap of the DTP core signatures enrichment in all NSCLC patient’s epithelial cells combined or split by disease status. The color scale represents the NES, and the stars indicate the significance of the adjusted *p* value. ∗*p* < 0.05vs. treatment-naive samples, determined using an adaptive multi-level split Monte-Carlo method. (H) Heatmap of the DTP core signatures enrichment in NSCLC patient’s epithelial cells according to the disease status and time of biopsy (early, <30 days since treatment initiation; late, >30 days). The color scale represents the NES, and the stars indicate the significance of the adjusted *p* value. ∗*p* < 0.05 and ∗∗*p* < 0.01 vs. treatment-naive samples, determined using an adaptive multi-level split Monte-Carlo method. (I) Analysis of the enrichment of the DTP core signature within minimal residual disease (MRD) transcriptional profiles.^50^ The color scale represents the NES, and the stars indicate the significance of the adjusted *p* value. ∗*p* < 0.05, ∗∗*p* < 0.01, and ∗∗∗*p* < 0.001 vs. treatment-naive samples, determined using an adaptive multi-level split Monte-Carlo method. EA, esophageal adenocarcinoma; BRCA, breast cancer adenocarcinoma; READ, rectal adenocarcinoma; COAD, colon adenocarcinoma; OV, ovarian cancer; GBM, glioblastoma; C/T, number of samples for the control/treated comparison; chemo, chemotherapy; ICI, immune checkpoint inhibitor; radio, radiotherapy.

Persister cell signature has been characterized across a range of experimental models and treatment regimens.^32^^,^^46^^,^^47^^,^^48^^,^^49^ Building upon this existing research, we compared hallmark enrichments upon persistence in published datasets with those identified in chemo-persistent A549 spheroids (Figures 3A and 3B). Pathways directly triggered by drug exposure, such as xenobiotic metabolism, p53 signaling, and apoptosis, showed variable enrichment depending on the model (Figure 3B). Similarly, processes associated with metabolic rewiring and EMT also differed in their activation status across datasets. However, enrichment of pathways in relation to proliferation (e.g., G2M checkpoint) were decreased in all datasets, in line with the reduced G2M proportion observed in the A549 spheroid model (Figures 3B and 2C).

Importantly, the most conserved upregulated pathway across persistent datasets was the hallmark “KRAS signaling down” (Figures 3B and S3D) corresponding to genes downregulated upon KRAS activation in various experimental settings.^51^^,^^52^^,^^53^^,^^54^ Consistently, mTORC1, a signaling pathway downstream of KRAS, as well as the processes it regulates, including glycolysis and cholesterol homeostasis, showed a consistent trend toward downregulation across datasets (Figures 3B and S3E). Thus, persistent models shared conserved features, despite heterogeneity between the experimental models, treatment regimens, or RNA sequencing methods.

Venn diagram analysis revealed that all six persistent datasets shared 126 significantly downregulated and 53 upregulated genes (Figures 3C and 3D; Table S6), forming the DTP up- and down-core persistence signature, respectively. To assess these sets of genes’ clinical relevance, we evaluated their enrichment in scRNA-seq data generated from NSCLC patients prior, during, and after TKIs.^12^ The DTP signatures in epithelial cells were not enriched upon treatment when considering the total pool of patients. However, grouping patients by their Response Evaluation Criteria in Solid Tumors (RECIST) at the time of the biopsy revealed that patients with a partial response or a stable disease were enriched for both DTP signatures, while patients with treatment resistance (progressive disease) were not (Figures 3E–3G). Note that, in line with the time frame used *in vitro*, this enrichment was more pronounced in biopsies sampled within the first 30 days of treatment (Figure 3H), suggesting that the identified DTP signature accounts for early persistent stages. We next assessed the robustness of the signatures in multiple solid cancers.^50^ This analysis showed that the DTP up- or down-core persistence signature is enriched upon treatment in 10 of the 17 bulk RNA-seq datasets, notably in breast, esophageal, and rectal cancers (Figure 3I). Taken together, these results indicate that the DTP core persistence signature is a tool to identify persistence in clinical settings.

### Oncolytic viruses as a potent therapeutic agent to target persister cells

**Figure 4:**
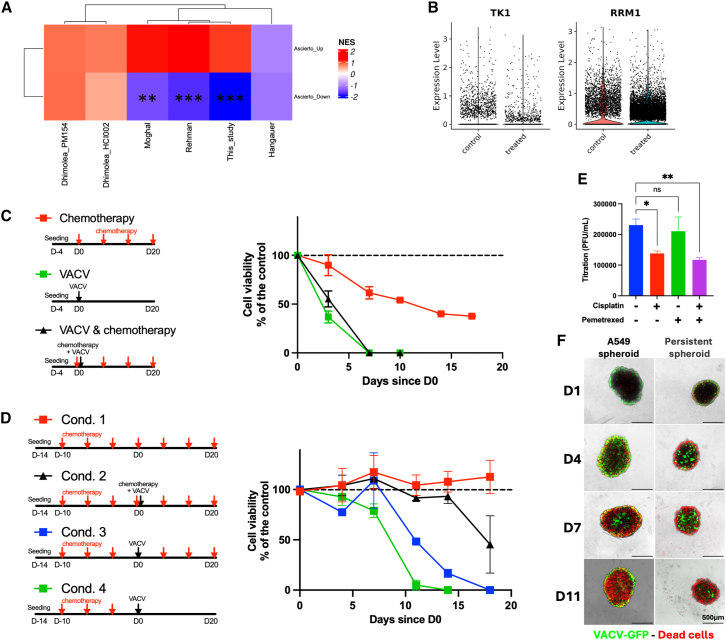
VACV efficacy in a persistent and treatment-free context (A) Enrichment of signatures associated with permissivity to VACV oncolytic viruses^55^ in DTP preclinical datasets. The *Ascierto_UP* and *Ascierto_DOWN* signatures correspond to genes up- and down-regulated in cells that are more permissive to VACV infection, respectively. The color scale represents the normalized enrichment score (NES), and the stars indicate the significance of the adjusted *p* value. ∗∗*p* < 0.01, and ∗∗∗*p* < 0.001 vs. treatment-naive condition, determined using an adaptive multi-level split Monte-Carlo method. (B) Violin plots depicting the levels of *TK1* and *RRM1* in the scRNA-seq dataset of control and chemo-persistent A549 spheroids. (C) Four days after seeding, A549 spheroids were treated with standard chemotherapy (10 μM cisplatin and 250 μM pemetrexed), the VACV at 2 × 10^5^ PFU/mL, or a combination of both agents for the indicated time. Cell viability was expressed as a percentage of the vehicle-treated control at matched time points. Nine spheroids per condition/time point, in three biological replicates; mean (SD). (D) Persistent spheroids were generated by exposure to 10 μM cisplatin and 250 μM pemetrexed of A549 spheroids for 10 days. Once persistent (D0), the efficacy of the VACV and the standard-of-care chemotherapy was determined following the indicated schedules (conditions 1–4). Viability was expressed as a percentage of vehicle-treated persistent spheroids at matched time points. Nine spheroids per condition/time point, in three biological replicates; mean (SD). (E) The VACV was incubated alone, in the presence of 10 μM cisplatin, 250 μM pemetrexed, or both for 3 h, and titration assays measuring plaque-forming unit (PFU) potency were performed. Three biological replicates; mean (SD); ns *p* ≥ 0.05, ∗*p* < 0.05 and ∗∗*p* < 0.01 vs. the control condition, determined using one-way ANOVA followed by a Tukey’s multiple comparisons test. (F) Representative images of GFP-expressing VACV and RFP-stained dead cells in A549 spheroids and persistent spheroids infected for the indicated time. Scale bar, 500 μm.

Interestingly, a deeper analysis of the various datasets revealed that the transcript levels of genes previously reported to negatively impact VACV efficacy^55^ are downregulated in several MRD and DTP datasets, including our A549 spheroid model (Figures 4A and S4A). However, the expression of those promoting VACV efficacy was similar upon treatment. This pattern suggests that the broad transcriptional downregulation induced during persistence incidentally suppresses programs restricting the OV infection. To experimentally challenge this hypothesis, we tested the efficacy of a modified VACV from the Copenhagen strain, bearing deletions of genes encoding the viral TK and the large RR subunit. These modifications increase its specificity for tumoral cells, thereby enhancing its safety profile.^30^^,^^56^ The modified VACV is thus reliant on the host cell expression of the gene encoding TK (*TK1*) and the large subunit RR (*RRM1*), both genes overexpressed in tumoral cells. Importantly, analysis of our transcriptomic data revealed that both *TK1* and *RRM1* remain expressed in a persistent context (Figure 4B).

First, the efficacy of VACV was evaluated in A549 spheroids. Control spheroids grown for 4 days were treated with chemotherapy (every 3–4 days), 2 × 10^5^ plaque-forming units (PFU)/mL VACV, or a combination of both. The concentration of the virus was chosen to match the theoretical circulating concentration expected in the clinic. Compared with chemotherapy, VACV alone was very efficient, with an undetectable ATP content 7 days post-virus addition (Figure 4C). The efficacy of the VACV/chemotherapy combination was similar to that of the VACV alone. Note that VACV alone was equally effective even at 100-fold lower concentrations (2.10^3^ PFU/mL) (Figure S4B), indicating that the initial viral dose is not a factor limiting the efficacy of the treatment in control spheroids.

Next, we determined the effect of the same treatment combinations on persistent spheroids generated after 10 days of chemotherapy, a stage at which they exhibit a significantly lower sensitivity to chemotherapy (Figure 1E). As expected, chemotherapy for 18 additional days did not reduce their viability (Figure 4D). In contrast, the addition of the VACV to chemotherapy reduced the viability to 43.8%, 18 days post-infection. To optimize VACV administration, alternative treatment schedules were investigated. Interrupting the chemotherapy treatment on the day of infection significantly enhanced the VACV efficacy, with the viability reaching zero 18 days post-infection. To gain insights into the reduced VACV efficiency in combination, we investigated the impact of chemotherapy on the DNA virus. The potency of the virus to form plaques during titration was reduced by 2-fold when pre-incubated with cisplatin for 3 h, while pemetrexed had no effect (Figure 4E). We then evaluated the effect of the virus alone on persistent spheroids. Strikingly, all tumoral cells were eliminated 14 days after a 2.10^5^ PFU/mL viral infection (Figure 4D). In line with these results, the signal of a fluorescent reporter (the green fluorescent protein, GFP) encoded in the VACV was similar in control and chemo-persistent spheroids, showing that the virus progresses from the edge toward the center of the spheroids, consequently triggering cell death in the same manner (Figure 4F). However, in contrast to control spheroids, a dose 100-fold lower showed slower and decreased killing kinetics in persistent spheroids (Figure S4C). Taken together, these results demonstrate that the VACV effectively eliminates both treatment-naive and persister cells, but the effect in persistent spheroids is dependent on the initial viral dose.

### Validation of the persistence signature and VACV efficacy in patient-derived organoids

**Figure 5:**
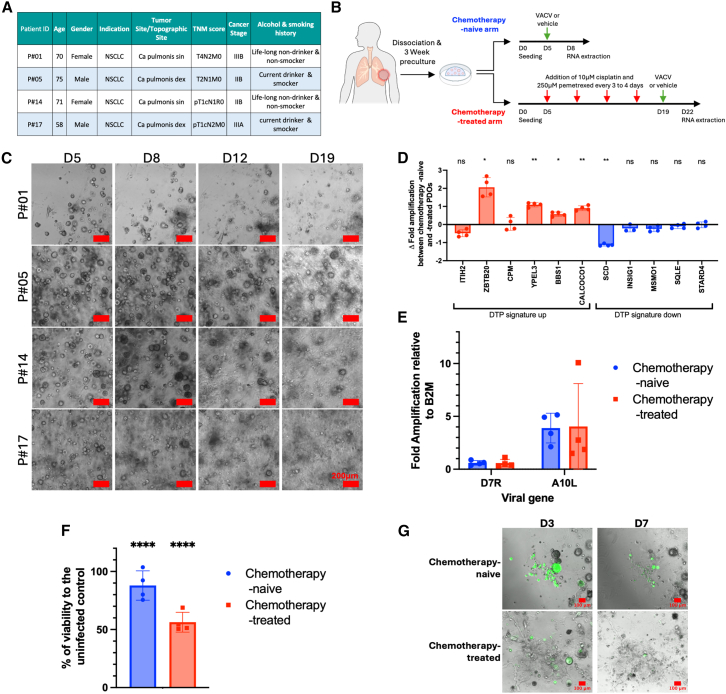
Persistence signature and VACV efficacy in patient-derived organoids (A) Characteristics of the patients and their tumor used to generate the patient-derived organoids (PDOs). (B) Schematic representation of the experimental protocol. (C) Representative bright-field images of treated PDOs at the indicated time. Scale bars, 200 μm. (D) Fold change of the transcript levels of genes associated with persistence between non-infected chemotherapy-treated PDOs (day 22 of the treated arm) and non-infected chemotherapy-naive PDOs (day 8 of the control arm). Each dot represents one patient; mean (SD); ns, *p* ≥ 0.05, ∗*p* < 0.05 and ∗∗*p* < 0.01 vs. chemotherapy-naive PDOs, determined using paired *t* tests. (E) Transcript levels of the early viral gene D7R and of the late viral gene A10L, 3 days post-infection, in chemotherapy-naive and chemotherapy-treated PDOs, determined by RT-qPCR. Each dot represents one patient. Data are presented as the mean (SD). (F) Viability 12 days post-infection in naive and chemotherapy-treated PDOs. The results are expressed as the percentage relative to the matched uninfected control. Each dot represents one patient, mean (SD); ∗∗∗∗*p* < 0.0001 vs. matched uninfected PDOs, determined using two-way ANOVA followed by a Šídák’s multiple comparisons test. (G) Representative merged bright-field and GFP-expressing VACV images of PDOs derived from patient #17 infected for the indicated time points. Scale bars, 100 μm.

To strengthen the validity of our finding beyond cell-line models, we investigated the effect of prolonged exposure to cisplatin and pemetrexed on PDOs. To this end, we isolated cells from non-squamous lung adenocarcinoma resections of treatment-naive patients (Figure 5A). Biopsy-derived cells from four patients were amplified for 3 weeks before the beginning of the experiment. The treatment protocol was similar to the one previously described for the A549, consisting of 250 μM pemetrexed combined with 10 μM cisplatin administered every 3–4 days for 2 weeks (Figure 5B). This protocol reduced the number of organoids and impaired growth in PDOs in a time-dependent manner (Figure 5C). Several transcript levels of top genes of the DTP signature identified above were deregulated in chemotherapy-treated compared with naive PDOs (Figure 5D), supporting a conserved transcriptional reprogramming across spheroids and PDO models. Finally, we assessed the effect of the VACV on naive or chemotherapy-treated PDOs. The proportion of GFP-positive cells from dissociated organoid domes recovered 3 days post-infection varied between 5.8% and 2% and decreased over time (Figures S5A and S5B), consistent with previous studies reporting that hydrogels can limit viral access to tumoral cells.^57^^,^^58^ Nevertheless, quantitative reverse-transcription PCR (RT-qPCR) analysis revealed the presence of *D7R* and *A10L* viral transcripts (Figure 5E), markers of early and late stages of the infection, respectively,^59^^,^^60^^,^^61^ indicating that the VACV fully completed its viral replication cycle in both naive and chemotherapy-treated PDOs. This infection ultimately led to oncolysis in both conditions, as shown by the significant decrease in viability (Figure 5F) and by the progressive disappearance of infected organoids over time, revealed by the expression of a viral fluorescent gene (Figure 5G).

## Discussion and conclusion

This study highlights the use of long-term cultured A549 spheroids as a robust *in vitro* model to investigate DTP mechanisms and evaluate therapeutic strategies. This platform enabled the profiling of chemotherapy-induced DTPs in NSCLC. Integrated with additional preclinical persistent datasets, we defined a core DTP signature conserved across multiple cancer subtypes and therapeutic strategies. Although derived from reductionist models, this signature was also enriched in patient-derived MRD samples, supporting its clinical relevance. Finally, this study uncovers the therapeutic potential of virotherapy in the context of NSCLC MRD, both in immortalized cell line spheroids and PDOs.

Prolonged chemotherapy treatment of the A549 3D spheroid NSCLC model induces a DTP state, effectively recapitulating MRD. Upon treatment, the model is characterized by a biphasic response: an initial decrease in viability followed by a plateau phase characterized by reduced proliferation and diminished drug sensitivity. Upon drug withdrawal, spheroids resumed proliferation and regained sensitivity, indicating that persistence was governed by reversible mechanisms. These characteristics align with the functional hallmarks of DTPs.^7^^,^^46^

Although not an oncogene per se, NRF2 acquires oncogenic functions in cancer contexts such as NSCLC.^62^ In A549 cells, KEAP1 is frequently inactivated through genetic and epigenetic mechanisms, contributing to sustained NRF2 activation.^63^^,^^64^ While KEAP1 state is not directly assessed in the present study, our results suggest a context-dependent activation (treatment or oxygen accessibility) of NRF2. Within control spheroids, the NRF2 signature was strongly downregulated in clusters with hypoxic signature, suggesting a localization at the spheroid core with poor oxygen access. On the other end, chemotherapy-treated spheroids transitioned into a transcriptionally homogeneous population characterized by activation of the NRF2 program, known to regulate antioxidant defense and metabolic adaptation.^65^ Clinically, high NRF2 expression has been observed in MRD and is associated with resistance and poor prognosis.^33^^,^^66^ NRF2 activation has also been reported in several preclinical studies exploring persistent phenotypes.^47^^,^^67^ Notably, Oren et al. demonstrated that inactivation of KEAP1 increased the proportion of proliferating DTPs.^11^ Therapeutic strategies aiming at targeting the NRF2-dependent metabolic state, including glutaminase inhibition, are currently under investigation in clinical trials in NSCLC.^68^^,^^69^ In addition, approaches targeting the regulation of ferroptosis,^49^ also mediated by the NRF2 program,^70^ may represent a promising strategy to target DTPs. However, validation of these strategies in more complex persistent models, as well as the assessment of potential off-target effects, remains necessary.^32^

Beside NRF2, direct comparison between treated and untreated spheroids revealed that cells adapt to treatment through targeted changes that directly impact drug efficacy, alongside broader metabolic changes. Upregulation of the drug-affected enzymes, drug inactivation and export mechanisms, and pathways alleviating the effects of both cisplatin and pemetrexed were observed. Beyond these specific changes, the proportion of cells in G2M was reduced, with an increase of cells in both S and G1 phases. The enrichment of G1-phase cells is a well-documented feature of persister cells, indicating a shift toward reduced proliferation.^7^^,^^11^^,^^46^ A proliferative state shift of cells in S phase is less commonly associated with persistence, but has been reported as an effect of pemetrexed treatment in A549 cells.^34^ A stalled S-phase may provide additional time for DNA repair, helping cells withstand treatment-induced stress. In parallel, treatment-induced adaptations included suppression of apoptosis, and a marked metabolic reprogramming from glycolysis to oxidative phosphorylation^71^ and fatty acid β-oxidation,^72^^,^^73^ hallmarks of DTP biology. Unexpectedly, hypoxia and EMT hallmarks were downregulated in treated spheroids despite their known association with persistence.^13^^,^^74^ This discrepancy might be linked to size differences between control and treated spheroids. Indeed, control spheroids showed a progressive increase in the expression of hypoxia-related genes over time and displayed a stronger hypoxic signature than the smaller treated spheroids. These observations are consistent with the formation of an oxygen gradient in control spheroids,^26^ influencing their transcriptomic profile. Furthermore, EMT, which can be induced in hypoxic conditions,^75^ was also enriched in hypoxia-high cells from control spheroids. These observations highlight a limitation of 3D models, as size-related effects may influence transcriptional deregulation when comparing treated and control conditions.

A549 cells harbor the KRAS mutation, which is the most common alteration in NSCLC cancers. This mutation leads to constitutive activation of KRAS, driving cancer initiation, proliferation, and survival.^76^ Notably, chemotherapy exposure resulted in downregulation of genes associated with KRAS and mTORC1 downstream activity, even in A549 cells bearing activating KRAS mutations. Similar transcriptional changes were observed in both NSCLC spheroids, PDOs and across multiple persistence datasets, suggesting a conserved reprogramming independent of the mutational context, treatment, or indication. However, while transcriptomic analysis informs on the expression of genes downstream of KRAS or mTOR signaling, it does not allow a clear determination of the activation or inhibition state of these pathways. Nevertheless, targeting the autophagy flux, enhanced following mTORC1 inhibition, has shown promising results in persistent colorectal cancer preclinical models^48^ and is currently being evaluated in clinical trials to target DTPs in patients (NCT05953350 and NCT05843188).

The conserved deregulation of several genes across preclinical datasets enabled the identification of pan-cancer signatures of persistence. To assess the clinical relevance of these signatures, we analyzed scRNA-seq data of NSCLC tumor samples collected at different time points during TKI treatment.^12^ The identified DTP signature was enriched in samples from patients with a stable disease or a treatment response, but not in resistant patients, consistent with the definition of persistence. This observation validates that the identified DTP signature does not simply reflect the exposure to treatment, but rather a cellular state distinct from resistance. Notably, the enrichment was the strongest in biopsies sampled early during treatment, suggesting that the persistence signature identified from preclinical models mainly reflects an early persistent state in patients. Despite differences in transcriptomic platforms, sequencing depth, and cell resolution, DTP signatures were also detectable in MRD bulk datasets, encompassing multiple indications and different treatment protocols.

Taken together, these results indicate that transcriptional changes observed in preclinical models are also detectable in patients undergoing treatment, prior to drug resistance. In preclinical models, DTPs have been described as a state that precedes and supports the emergence of a stable resistance.^8^^,^^77^^,^^78^ However, whether the degree of persistence enrichment in MRD samples can predict resistance development in patients remains to be determined.

Nevertheless, the presence of a conserved persistence signature across multiple indications and treatment contexts highlights the potential broad applicability of strategies targeting persistence.

The transcriptomic signature of several persistent datasets suggested a potential increased susceptibility to oncolytic virotherapy. To evaluate this, a TK^−^/RR^−^ VACV, dependent on host *TK1* and *RRM1* expression—both retained in the persistent state—was tested. This virus was selected for its engineered safety profile and specificity to tumoral cells,^30^^,^^56^ and its natural immune evasion capabilities,^79^ given that DTP states have been associated with elevated inflammatory and interferon signaling.^80^^,^^81^^,^^82^

While tested concentrations of the VACV-chemotherapy combinations yielded similar efficacy to the VACV alone in control spheroids, concomitant administration of chemotherapy significantly reduced the efficacy of the VACV in the persistent context. This reduction likely reflects the direct impairment of the virus by chemotherapy, and a decreased viral efficiency within cells in a state of persistence. In this context, the starting virus concentration influences VACV efficacy, with lower doses delaying the loss of spheroid viability. Mechanistically, exposure to cisplatin reduced the number of functional viral particles, likely due to its ability to form adducts on the viral DNA, thereby interfering with its replication. While further validation is required to fully elucidate the negative impact of cisplatin on the VACV, it highlights the need for caution when designing combination therapies. Nevertheless, as a standalone strategy, the VACV retained antitumor activity, supporting efficient viral replication and tumor cell elimination in NSCLC spheroid and PDO models, across both control and persistent contexts.

Validating VACV efficacy in additional NSCLC subtypes, including squamous cell carcinoma models, and harboring distinct oncogenic drivers would be of interest. In addition, using models incorporating an immune compartment will be required to assess whether the tumor cell-intrinsic susceptibility to the VACV observed *in vitro* is maintained within a more complex tumor microenvironment. This is especially relevant given that the persistent TME has been described as immunosuppressive,^80^^,^^83^^,^^84^ while the VACV is known to re-engage the immune system against tumor cells.^85^^,^^86^ Preclinical tests of administration could be valuable as well, as multiple routes can be considered for OV treatment in NSCLC, including intra-tumoral, -venous, -arterial, and -pleural administration,^87^^,^^88^ with the aim of enhancing the targeting of disseminated lesions or rather promoting viral persistence within the tumor.

While transcriptomic analyses have guided the development of strategies to eliminate persister cells, many of these approaches target broadly conserved pathways, raising concerns about toxicity when administered systemically. The VACV offers a selective alternative, particularly if engineered to locally express transgenes targeting persistence-specific adaptations, such as NRF2 signaling, ferroptosis regulation, or autophagy. Such a two-pronged approach, combining tumor-specific oncolysis with localized therapeutic expression, could enhance persister cells elimination while minimizing off-target effects. As such, virotherapy represents a promising avenue to target MRD, with the aim of preventing relapse across solid tumors.

## Material and methods

### Cell lines and cell culture

The human lung cancer cell line A549, was obtained from the American Type Culture Collection (ATCC, VA, USA, CCL-185), tested for mycoplasma, and grown in DMEM (Gibco, MA, USA, D6429) supplemented with 10% fetal bovine serum (FBS; Corning, NY, USA, 35-070-CV) and 5% gentamycin sulfate 4 g/L (Sigma-Aldrich, MO, USA, G1272). Cells were incubated at 37°C in a 5% CO_2_ atmosphere. For 3D cultures, cells were seeded at a density of 10,000 cells/well in a 96-well Ultra-Low Attachment (ULA) plate (Thermo Fisher Scientific, MA, USA, Nunclon Sphera, 174925) with 200 μL of media. Half of the media was renewed twice weekly. When needed, dissociation of spheroids into single cells was performed by pooling spheroids together, washing with PBS (Sigma-Aldrich, MO, USA, D8537), and 20-min incubation at 37°C in Accutase (Sigma-Aldrich, MO, USA, SCR005) with pipetting every 5 min.

### Human resection sample dissociation and cryopreservation

Human specimens were obtained from the company Fidelis Research AD, which provides biological samples collected under Ethics approval and informed consent for research purposes. Upon receipt, fresh resection samples were washed in PBS (Sigma-Aldrich, MO, USA, D8537) to remove blood and minced with scissors. Samples were then enzymatically digested using the Human Tumor Dissociation Kit (Miltenyi Biotech, Germany, 130-095-929) in C tubes (Miltenyi Biotech, Germany, 130-093-237) with the gentleMACSTM Octo dissociator (Miltenyi Biotech, Germany). The content of the C tube was then filtered through a 70-μm cell strainer. Strained cells were centrifuged, then resuspended in 1 mL lysis buffer (Sigma-Aldrich, MO, USA, R7757), and incubated 5 min at room temperature (RT), to remove the remaining blood cells. Cells were then washed with PBS, pelleted, and resuspended in PBS for counting. Vials of cells were cryopreserved using the Bambanker cryopreservation media (NIPPON GENETICS, Japan, BB05) according to manufacturer’s instructions.

### PDO culture

Patient cells were thawed from cryopreserved vials and seeded in 50 μL domes of 50% Cultrex RGF BME, type 2 (R&D systems, MN, USA, 3043535.00) at a concentration of 250,000 cells per dome in 24-well plates (TPP, Switzerland, 92024). For passaging during the preculture and subsequent experiments, seeding patient cells concentration was set at 20,000 cells per 50-μL domes. The cells were cultured at 37°C in a 5% CO_2_ atmosphere, in Advanced DMEM/F12 (Gibco, MA, USA, 12634-010) supplemented with B27 (Gibco, MA, USA, 17504-44), N2 (Gibco, MA, USA, 1752-048), 2 mM GlutaMAX (Gibco, MA, USA, 35050-38), 10 mM HEPES (Sigma-Aldrich, MO, USA, H0887), 1% FBS (Sigma-Aldrich, MO, USA, P4417-50TAB), 100 μg/mL penicillin-streptomycin (Gibco, MA, USA, 15140-122), 1.25 nM N-acetyl-L-cysteine (Sigma-Aldrich, MO, USA, A7250), 10 ng/mL of FGF-10 (STEMCELL Technologies, Canada, 78037.1), and 5 ng/mL EGF (STEMCELL Technologies, Canada, 78136). Half of the media was refreshed twice weekly. 10 μM Y27632 (STEMCELL Technologies, Canada, 72302) was added to the media after each passaging. During passaging, the media was removed from wells, replaced with 300 μL of Dispase (STEMCELL Technologies, Canada, 07923), then incubated for 20 min at 37°C. The domes were then disrupted by pipetting. The resulting solution was pooled into a falcon filled with PBS (Sigma-Aldrich, MO, USA, D8537) and centrifuged at 500 × *g* for 5 min at 4°C. The pellet was then resuspended in TrypLE Select (Gibco, MA, USA, 12563011) and incubated at 37°C for 10–15 min with pipetting every 5 min, until obtention of a single-cell suspension. During the 3-week preculture phase, organoids were passaged once, after 2 weeks of culture. Post-treatment, PDOs were re-seeded in fresh extracellular matrix by incubating the domes for 20 min at 37°C with 300 μL of Dispase, washing with PBS, and then re-seeding in 50 μL Cultrex domes at the same initial density.

### Chemotherapy treatments

Stock solutions of 1 mM cisplatin (Sigma-Aldrich, MO, USA, PHR1624) and 30 mM pemetrexed (Sigma-Aldrich, MO, USA, PHR1596) in 0.9% NaCl (Merck, Germany, 16224) were stored at −20°C. The protocol inducing persistence started 4 days post-spheroid’s seeding. The chemotherapy was added twice weekly, during media changes, where half of the media was replaced with fresh media containing chemotherapy at a final concentration of 10 μM cisplatin and 250 μM pemetrexed per well. The control condition was treated with the vehicle (0.9% NaCl solution). To assess the sensitivity of spheroids to the chemotherapy over time, control and treated spheroids were harvested at different culture times and were treated with either vehicle or a high bolus of cisplatin and pemetrexed (100 μM and 2,500 μM, respectively) during media change. To study the regrowth during drug holiday, control and treated spheroids were first dissociated after 14 days of culture and re-seeded at a concentration of 10,000 cells/well in a 96-well ULA plate and then cultured without treatment with media changes twice a week. Once proliferation resumed in both conditions, they were treated with 10 μM cisplatin and 250 μM pemetrexed.

### VACV generation and infection

The VACV, a double-deleted TK^−^RR^-^ VACV expressing the GFP, was constructed and characterized previously.^89^ VACV was produced in chicken embryo fibroblasts and titrated by plaque assay on Vero cells (ATTC, VA, USA, CCL-81). The infection of spheroids was performed during media changes where half the media was replaced with fresh media containing the OV, for a final concentration of 2 × 10^5^ PFU/mL per well.

### VACV: Chemotherapy interaction test

2 × 10^5^ PFU/mL VACV was incubated for 3 h at 37°C in a 5% CO_2_ atmosphere either in the A549 media alone, supplemented with 10 μM cisplatin, supplemented with 250 μM pemetrexed, or with both chemotherapies (see the “chemotherapy treatments” section for the drug preparation protocol). The virus was then pelleted by centrifugation at 5,000 × *g* overnight at 4°C and resuspended in PBS (Sigma-Aldrich, MO, USA, D8537). Viral titration was performed by plaque assay on Vero cells (ATTC, VA, USA, CCL-81).

### Cell viability assays

#### ATP content

The amount of ATP present in individual spheroid was determined using the CellTiter-Glo 3D kit (Promega, WI, USA, #G9681). Spheroids were transferred from the ULA culture plate into white-opaque 96-well plates (Corning, NY, USA, 3355), in 50 μL of culture media and 50 μL of CellTiter-Glo 3D reagent. Plates were shaken for 5 min followed by 25 min of incubation in the dark at RT. The signal was recorded using a microplate reader (Tecan, Switzerland, Spark Multimode Microplate Reader), with a settle time of 50 ms and an integration time of 500 ms. ATP concentration was quantified using a calibration curve based on rATP standards (Promega, WI, USA, P1132). For each condition, the viability of three spheroids was measured, and the average value and standard deviation were calculated.

#### Spheroid surface monitoring

Bright-field images were taken with the Eclipse Ti (Nikon, Japan), at different culture times. The surface was quantified using the ImageJ software (v.1.54j).

#### VACV infection imaging

To follow cell death upon infection, Incucyte Cytotox Red Dye (Sartorius, Germany, 4632) was added at a final concentration of 2.5 mM/well. The dye was added again at each media change or treatment. Images were acquired at least 2 h after dye addition. Fluorescent images were taken with the Leica Thunder Imager Live cell & 3D assay . Spheroids’ images were computationally cleared using LAS X software. Maximum intensity projections and GFP signal profiles were generated from 5 z-planes, using the ImageJ software (v.1.54j).

#### FACS

PDOs were dissociated into single cell using the protocol for the passage. The cells were washed twice with PBS and then filtered using the 40-μm cell filters (Merck, Germany, BAH136800040). Single cells were incubated with a near-infrared LIVE/DEAD staining (Invitrogen, CA, USA, L10119) diluted at 1:1,500 for 30 min, protected from light. Samples were analyzed by a MACSQuant Analyzer 16 Flow Cytometer (Miltenyi Biotech, Germany) and the Kaluza software. This analysis enabled the quantification of GFP-positive cells and dead cells.

### RT-qPCR

Total RNA was isolated from dissociated PDOs with the RNeasy Plus Micro Kit (QIAGEN, Netherlands, 74034), according to the supplier’s protocol. RNAs were quantified by spectrophotometry (NanoDrop, Thermo Fisher Scientific, MA, USA). For RT-qPCRs targeting human genes, 500 ng of total RNA was used to synthetize cDNA using random hexamers (Thermo Fisher Scientific, MA, USA, SO132) and the SuperScript IV reverse transcriptase kit (Thermo Fisher Scientific, MA, USA, 18090010), following the manufacturer’s instructions. Quantitative PCR reactions were performed using the Light Cycler 480 SYBR Green I Master ×2 Kit (Roche, Switzerland, 04887352001) and the CFX Opus 384 (Bio-Rad, CA, USA), according to the supplier’s protocol, and relative cDNAs concentrations were quantified by a standard curve method. To quantify the transcript level of *A10L* and *D7R* genes, 50 ng of total RNA was used to perform the reverse transcription and quantitative PCR reactions using Master Mix TaqPath One-Step RT-qPCR TaqPath (Applied Biosystems, MA, USA, A15300) and the QuantStudio 3 (Thermo Fisher Scientific, MA, USA) according to the supplier’s protocol. Transcripts from the housekeeping gene *B2M* were amplified and probed using the kit Human B2M Endogenous Control (Applied Biosystems, MA, USA, 4326319E). Primer and probes are listed in Table S7.

### Single cell RNA-seq

#### Sample preparation analysis

Spheroids under chemotherapy treatment for 14 days and time-matched control in three biological replicates were dissociated and filtrated using 40-μm cell trainers (Bel-Art, NJ, USA., H13680-0040) to avoid cell clumps. Samples were fixed following instructions of the manufacturer (Chromium Next GEM Single Cell Fixed RNA Sample Preparation Kit, 10× Genomics, CA, USA, 1000414) and stored at −80°C. Up to 2 million cells were processed per hybridization following 10× Genomics recommendations. For each experiment, we pooled an equal number of cells from each hybridization to have an equal contribution per sample. The samples were processed on the Chromium Controller from 10× Genomics. The sequencing was performed on the NovaSeq X platform (Illumina, CA, USA), with a target depth of 30,000 reads per cell. Filtered matrices were generated using CellRanger v.7.1.0. The single-cell library preparation, sequencing, and preprocessing were performed by Single Cell Discoveries. Data analysis using the Seurat R package v.5.2.1 was conducted on R studio (v.2022.12.1) using R version 4.3.2 (2020-10-10).

#### Pre-processing

Cells with more than 200 expressed genes and with less than 10% of mitochondrial genes were used to generate a single Seurat object containing the biological replicates of the treated and control conditions (Figure S1A). One biological replicate from the control condition was excluded due to aberrant signature. After log normalization (scale factor, 10,000), the dataset was integrated using the harmony package^90^ 1.2.3 (RunHarmony) with the biological replicate variable as the integration factor to reduce batch effects between replicates (Figure S1B). The Harmony-corrected embeddings were used to construct a nearest-neighbor graph (using the first 20 harmony dimensions), and clustering was performed at a resolution of 0.5. For visualization, the UMAP was computed based on the first 20 dimensions of the harmony embeddings. Cell cycle scores were determined using the Seurat package. The deregulated genes between treated and control conditions were identified using the FindMarkers function. The markers for each cluster were generated by the FindAllMarkers function.

#### Trajectory analysis

Single-cell trajectory analysis was performed (dyno package 0.1.2)^91^ using the slingshot method^92^ to reconstruct lineage relationships and infer pseudotime ordering of cells. Slingshot was run with default parameters without any root. Pseudotime values and inferred lineages were used to visualize gene expression dynamics along trajectories and to identify genes whose expression changes significantly along pseudotime (important features).

#### GSEA

Pathway analyses were performed with the R package fgsea 1.28.0. To compare treated versus control conditions, or clusters between each other, a gene list was used containing deregulated genes expressed in at least 30% of cells and ranked by fold change. The default parameter of permutations numbers (nPerm) was used in all the analysis except for analysis of literature DTP datasets, where it was increased to 1 × 10^9^ to ensure robust estimation of *p* values and enrichment significance in datasets with unbalanced and wider gene expression values. The enrichment plot was made with the function plotEnrichment using ranked by fold change or by feature importance from the trajectory analysis. The signatures' area under the curve (AUC) scores plotted on UMAPs were computed using the AUCell package. The NRF2 signature was obtained from the publication of Namani et al. in 2017,^33^ the cancer stem cell signature from Herrero-Pomares et al., 2019,^93^ and the signature associated with VACV permissivity from Ascierto et al., 2011.^55^ All the gene signatures used in this study are provided in Table S8.

#### PubMed data mining

Genes associated with persistence or resistance were identified by a systematic search on PubMed using the query: [GENE NAME] AND cancer AND (chemoresistance OR drug resistance) for the resistance search, and [GENE NAME] AND cancer AND (drug persistence OR drug tolerant persister cells OR DTP OR drug tolerance) for the persistence search. The gene and other keywords were searched in the titles and abstracts. The list of genes queried corresponds to genes of the single-cell dataset universe. Then, a Venn diagram was generated to identify among significantly deregulated genes upon treatment (adjusted *p*-value <0.05), genes previously associated with persistence or resistance in literature.

#### Comparison with literature

The fold change and *p* value from DTP preclinical studies were obtained from supplemental information from the publication of Dhimolea et al., 2021,^46^ Rehman et al., 2021,^48^ Hangauer et al., 2017,^49^ and from the GEO2R analysis (GSE198672). The DTP up- and down-core signatures correspond to genes significantly deregulated (*p* < 0.05) with a fold change greater than 0.1 across all preclinical DTP datasets. The NSCLC patient dataset was obtained from the publication of Maynard et al., 2020 and prepossessed following the publication pipeline before analysis.^12^ The fold change of MRD studies were obtained from the publication of Liu et al., 2024.^50^

### Statistical analysis

Comparisons between two groups were performed using Student’s *t* test; those between multiple time points, using one-way analysis of variance (ANOVA); and those between two groups across time, using two-way ANOVA. ANOVAs were followed by appropriate post hoc tests. Enrichment-adjusted *p* values were computed using the fgsea package with the adaptive multi-level split Monte-Carlo method. *p* values < 0.05, 0.01, 0.001, and 0.0001 were indicated by ∗, ∗∗, ∗∗∗, and ∗∗∗∗ respectively, whereas *p* values ≥ 0.05 were depicted as ns (not significant). The number of biological replicates included per condition is indicated in the figure legends.

## Data and code availability

The scRNA-seq dataset generated during this study is available on the EMBL-EBI Single Cell Expression Atlas platform: https://www.ebi.ac.uk/gxa/sc/experiments/E-ANND-7/results/cell-plots and on the https://provitd.github.io/studies website. The code used to generate the results of this study can be found on GitHub at https://github.com/provitd/A549_spheroid_DTP_2026.

## Acknowledgments

The authors thank Oriol Llorà-Batlle and Single Cell Discoveries for the processing of the samples, Valentine Gilbart for her advice on the data analysis, and Anil Shantilal Thanki, Iris Diana Yu, and Liora Vilmovsky for their help to upload datasets on the EMBL-EBI Single Cell Expression Atlas platform. The PERSIST-SEQ project has received funding from the 10.13039/501100010767Innovative Medicines Initiative 2 (www.imi.europa.eu) Joint Undertaking under grant agreement no 101007937. This Joint Undertaking receives support from the European Union’s Horizon 2020 research and innovation program and 10.13039/100013322EFPIA. The research was also funded by the 10.13039/501100001665French National Research Agency (ANR) through the Programme d'Investissement d'Avenir under contract ANR-10-LABX-0030-INRT grant under the frame programme Investissement d’Avenir ANR-10-IDEX-0002-02, as well as by the Interdisciplinary Thematic Institute IMCBio, as part of the ITI 2021-2028 program of the 10.13039/501100003768University of Strasbourg, 10.13039/501100004794CNRS, and 10.13039/501100001677Inserm; by IdEx Unistra (ANR-10-IDEX-0002); and by SFRI-STRAT’US project (ANR 20-SFRI-0012) and EUR IMCBio (ANR-17-EURE-0023) under the framework of the French Investments for the Future Program. C.F. was supported by the Ministry of Higher Education and Research and INCa 18498.

## Author contributions

Conceptualization, C.F., G.L., S.J., J.-M.B., E.L., and P.E.; investigation, C.F., E.L., B.M., and S.C.; data analysis, C.F. and J.D.; writing – original and revised draft, C.F., S.J., and G.L.; writing – review and editing, J.-M.B. and P.E.; supervision, S.J. and G.L.

## Declaration of interests

C.F., S.J., S.C. and P.E. have been employees and are shareholders of Transgene. E.L. and J.-M.B. were employees of Transgene S.A. at the time of the study design and early experimental studies.

This communication reflects the views of the PERSIST-SEQ consortium and neither IMI nor the European Union and EFPIA are liable for any use that may be made of the information contained herein.

## Supplemental information

Data S1. Tables S1–S8

Document S2. Article plus supplemental information

## References

[1] Wang X., Zhang H., Chen X. (2019). Drug resistance and combating drug resistance in cancer. Cancer Drug Resist..

[2] Chaudhuri A.A., Chabon J.J., Lovejoy A.F., Newman A.M., Stehr H., Azad T.D., Khodadoust M.S., Esfahani M.S., Liu C.L., Zhou L. (2017). Early Detection of Molecular Residual Disease in Localized Lung Cancer by Circulating Tumor DNA Profiling. Cancer Discov..

[3] Abbosh C., Rosenthal R., Birkbak N.J., Wilson G.A., Jamal-Hanjani M., Constantin T., Salari R., Le Quesne J., Moore D.A., Veeriah S. (2017). Phylogenetic ctDNA analysis depicts early-stage lung cancer evolution. Nature.

[4] Fennell K.A., Vassiliadis D., Lam E.Y.N., Martelotto L.G., Balic J.J., Hollizeck S., Weber T.S., Semple T., Wang Q., Miles D.C. (2022). Non-genetic determinants of malignant clonal fitness at single-cell resolution. Nature.

[5] Awad M.M., Liu S., Rybkin I.I., Arbour K.C., Dilly J., Zhu V.W., Johnson M.L., Heist R.S., Patil T., Riely G.J. (2021). Acquired Resistance to KRAS^G12C^ Inhibition in Cancer. N. Engl. J. Med..

[6] Sequist L.V., Waltman B.A., Dias-Santagata D., Digumarthy S., Turke A.B., Fidias P., Bergethon K., Shaw A.T., Gettinger S., Cosper A.K. (2011). Genotypic and Histological Evolution of Lung Cancers Acquiring Resistance to EGFR Inhibitors. Sci. Transl. Med..

[7] Sharma S.V., Lee D.Y., Li B., Quinlan M.P., Takahashi F., Maheswaran S., McDermott U., Azizian N., Zou L., Fischbach M.A. (2010). A Chromatin-Mediated Reversible Drug-Tolerant State in Cancer Cell Subpopulations. Cell.

[8] Ramirez M., Rajaram S., Steininger R.J., Osipchuk D., Roth M.A., Morinishi L.S., Evans L., Ji W., Hsu C.-H., Thurley K. (2016). Diverse drug-resistance mechanisms can emerge from drug-tolerant cancer persister cells. Nat. Commun..

[9] Sun X., Wu L.F., Altschuler S.J., Hata A.N. (2024). Targeting therapy-persistent residual disease. Nat. Cancer.

[10] Siegel R.L., Giaquinto A.N., Jemal A. (2024). Cancer statistics, 2024. CA Cancer J. Clin..

[11] Oren Y., Tsabar M., Cuoco M.S., Amir-Zilberstein L., Cabanos H.F., Hütter J.-C., Hu B., Thakore P.I., Tabaka M., Fulco C.P. (2021). Cycling cancer persister cells arise from lineages with distinct programs. Nature.

[12] Maynard A., McCoach C.E., Rotow J.K., Harris L., Haderk F., Kerr D.L., Yu E.A., Schenk E.L., Tan W., Zee A. (2020). Therapy-Induced Evolution of Human Lung Cancer Revealed by Single-Cell RNA Sequencing. Cell.

[13] Aissa A.F., Islam A.B.M.M.K., Ariss M.M., Go C.C., Rader A.E., Conrardy R.D., Gajda A.M., Rubio-Perez C., Valyi-Nagy K., Pasquinelli M. (2021). Single-cell transcriptional changes associated with drug tolerance and response to combination therapies in cancer. Nat. Commun..

[14] Lusky M., Erbs P., Foloppe J., Acres R.B. (2010). Oncolytic vaccinia virus: a silver bullet?. Expert Rev. Vaccines.

[15] Foloppe J., Kintz J., Futin N., Findeli A., Cordier P., Schlesinger Y., Hoffmann C., Tosch C., Balloul J.-M., Erbs P. (2008). Targeted delivery of a suicide gene to human colorectal tumors by a conditionally replicating vaccinia virus. Gene Ther..

[16] Azar F., Deforges J., Demeusoit C., Kleinpeter P., Remy C., Silvestre N., Foloppe J., Fend L., Spring-Giusti C., Quéméneur E., Marchand J.B. (2024). TG6050, an oncolytic vaccinia virus encoding interleukin-12 and anti-CTLA-4 antibody, favors tumor regression via profound immune remodeling of the tumor microenvironment. J. Immunother. Cancer.

[17] Lin D., Shen Y., Liang T. (2023). Oncolytic virotherapy: basic principles, recent advances and future directions. Signal Transduct. Targeted Ther..

[18] Todo T., Ito H., Ino Y., Ohtsu H., Ota Y., Shibahara J., Tanaka M. (2022). Intratumoral oncolytic herpes virus G47Δ for residual or recurrent glioblastoma: a phase 2 trial. Nat. Med..

[19] Andtbacka R.H.I., Collichio F., Harrington K.J., Middleton M.R., Downey G., Ӧhrling K., Kaufman H.L. (2019). Final analyses of OPTiM: a randomized phase III trial of talimogene laherparepvec versus granulocyte-macrophage colony-stimulating factor in unresectable stage III–IV melanoma. J. Immunother. Cancer.

[20] Goad D.W., Bressy C., Holbrook M.C., Grdzelishvili V.Z. (2022). Acquired chemoresistance can lead to increased resistance of pancreatic cancer cells to oncolytic vesicular stomatitis virus. Mol. Ther. Oncolytics.

[21] Weiland T., Lampe J., Essmann F., Venturelli S., Berger A., Bossow S., Berchtold S., Schulze-Osthoff K., Lauer U.M., Bitzer M. (2014). Enhanced killing of therapy-induced senescent tumor cells by oncolytic measles vaccine viruses. Int. J. Cancer.

[22] Sakhawat A., Ma L., Muhammad T., Khan A.A., Chen X., Huang Y. (2019). A tumor targeting oncolytic adenovirus can improve therapeutic outcomes in chemotherapy resistant metastatic human breast carcinoma. Sci. Rep..

[23] Monks A., Scudiero D., Skehan P., Shoemaker R., Paull K., Vistica D., Hose C., Langley J., Cronise P., Vaigro-Wolff A. (1991). Feasibility of a High-Flux Anticancer Drug Screen Using a Diverse Panel of Cultured Human Tumor Cell Lines. J. Natl. Cancer Inst..

[24] Kimlin L.C., Casagrande G., Virador V.M. (2013). In vitro three-dimensional (3D) models in cancer research: An update. Mol. Carcinog..

[25] Baker B.M., Chen C.S. (2012). Deconstructing the third dimension – how 3D culture microenvironments alter cellular cues. J. Cell Sci..

[26] Zanoni M., Piccinini F., Arienti C., Zamagni A., Santi S., Polico R., Bevilacqua A., Tesei A. (2016). 3D tumor spheroid models for in vitro therapeutic screening: a systematic approach to enhance the biological relevance of data obtained. Sci. Rep..

[27] Minchinton A.I., Tannock I.F. (2006). Drug penetration in solid tumours. Nat. Rev. Cancer.

[28] Wartenberg M., Ling F.C., Müschen M., Klein F., Acker H., Gassmann M., Petrat K., Pütz V., Hescheler J., Sauer H. (2003). Regulation of the multidrug resistance transporter P-glycoprotein in multicellular tumor spheroids by hypoxia-inducible factor-1 and reactive oxygen species. FASEB J..

[29] Jubelin C., Muñoz-Garcia J., Griscom L., Cochonneau D., Ollivier E., Heymann M.-F., Vallette F.M., Oliver L., Heymann D. (2022). Three-dimensional in vitro culture models in oncology research. Cell Biosci..

[30] Foloppe J., Kempf J., Futin N., Kintz J., Cordier P., Pichon C., Findeli A., Vorburger F., Quemeneur E., Erbs P. (2019). The Enhanced Tumor Specificity of TG6002, an Armed Oncolytic Vaccinia Virus Deleted in Two Genes Involved in Nucleotide Metabolism. Mol. Ther. Oncolytics.

[31] Dickgreber N.J., Fink T.H., Latz J.E., Hossain A.M., Musib L.C., Thomas M. (2009). Phase I and Pharmacokinetic Study of Pemetrexed plus Cisplatin in Chemonaive Patients with Locally Advanced or Metastatic Malignant Pleural Mesothelioma or Non–Small Cell Lung Cancer. Clin. Cancer Res..

[32] Russo M., Chen M., Mariella E., Peng H., Rehman S.K., Sancho E., Sogari A., Toh T.S., Balaban N.Q., Batlle E. (2024). Cancer drug-tolerant persister cells: from biological questions to clinical opportunities. Nat. Rev. Cancer.

[33] Namani A., Cui Q.Q., Wu Y., Wang H., Wang X.J., Tang X. (2017). NRF2-regulated metabolic gene signature as a prognostic biomarker in non-small cell lung cancer. Oncotarget.

[34] Giovannetti E., Mey V., Nannizzi S., Pasqualetti G., Marini L., Del Tacca M., Danesi R. (2005). Cellular and Pharmacogenetics Foundation of Synergistic Interaction of Pemetrexed and Gemcitabine in Human Non–Small-Cell Lung Cancer Cells. Mol. Pharmacol..

[35] Takezawa K., Okamoto I., Okamoto W., Takeda M., Sakai K., Tsukioka S., Kuwata K., Yamaguchi H., Nishio K., Nakagawa K. (2011). Thymidylate synthase as a determinant of pemetrexed sensitivity in non-small cell lung cancer. Br. J. Cancer.

[36] Chattopadhyay S., Moran R.G., Goldman I.D. (2007). Pemetrexed: biochemical and cellular pharmacology, mechanisms, and clinical applications. Mol. Cancer Therapeut..

[37] Amable L. (2016). Cisplatin resistance and opportunities for precision medicine. Pharmacol. Res..

[38] Li T., Zhu K., Tong H., Sun Y., Zhu J., Qin Z., Chen J., Wu L., Zhang X., Wang A. (2025). Cancer-associated fibroblast derived CXCL14 drives cisplatin chemoresistance by enhancing nucleotide excision repair in bladder cancer. J. Exp. Clin. Cancer Res..

[39] Arora S., Kothandapani A., Tillison K., Kalman-Maltese V., Patrick S.M. (2010). Downregulation of XPF–ERCC1 enhances cisplatin efficacy in cancer cells. DNA Repair.

[40] Zamagni A., Pasini A., Pirini F., Ravaioli S., Giordano E., Tesei A., Calistri D., Ulivi P., Fabbri F., Foca F. (2020). CDKN1A upregulation and cisplatin-pemetrexed resistance in non-small cell lung cancer cells. Int. J. Oncol..

[41] Li Y., Chen H., Xie X., Yang B., Wang X., Zhang J., Qiao T., Guan J., Qiu Y., Huang Y.-X. (2023). PINK1-Mediated Mitophagy Promotes Oxidative Phosphorylation and Redox Homeostasis to Induce Drug-Tolerant Persister Cancer Cells. Cancer Res..

[42] Goldman A., Khiste S., Freinkman E., Dhawan A., Majumder B., Mondal J., Pinkerton A.B., Eton E., Medhi R., Chandrasekar V. (2019). Targeting tumor phenotypic plasticity and metabolic remodeling in adaptive cross-drug tolerance. Sci. Signal..

[43] Arasada R.R., Shilo K., Yamada T., Zhang J., Yano S., Ghanem R., Wang W., Takeuchi S., Fukuda K., Katakami N. (2018). Notch3-dependent β-catenin signaling mediates EGFR TKI drug persistence in EGFR mutant NSCLC. Nat. Commun..

[44] Guler G.D., Tindell C.A., Pitti R., Wilson C., Nichols K., KaiWai Cheung T., Kim H.-J., Wongchenko M., Yan Y., Haley B. (2017). Repression of Stress-Induced LINE-1 Expression Protects Cancer Cell Subpopulations from Lethal Drug Exposure. Cancer Cell.

[45] Viale A., Pettazzoni P., Lyssiotis C.A., Ying H., Sánchez N., Marchesini M., Carugo A., Green T., Seth S., Giuliani V. (2014). Oncogene ablation-resistant pancreatic cancer cells depend on mitochondrial function. Nature.

[46] Dhimolea E., De Matos Simoes R., Kansara D., Al’Khafaji A., Bouyssou J., Weng X., Sharma S., Raja J., Awate P., Shirasaki R. (2021). An Embryonic Diapause-like Adaptation with Suppressed Myc Activity Enables Tumor Treatment Persistence. Cancer Cell.

[47] Moghal N., Li Q., Stewart E.L., Navab R., Mikubo M., D’Arcangelo E., Martins-Filho S.N., Raghavan V., Pham N.-A., Li M. (2023). Single-Cell Analysis Reveals Transcriptomic Features of Drug-Tolerant Persisters and Stromal Adaptation in a Patient-Derived EGFR-Mutated Lung Adenocarcinoma Xenograft Model. J. Thorac. Oncol..

[48] Rehman S.K., Haynes J., Collignon E., Brown K.R., Wang Y., Nixon A.M.L., Bruce J.P., Wintersinger J.A., Singh Mer A., Lo E.B.L. (2021). Colorectal Cancer Cells Enter a Diapause-like DTP State to Survive Chemotherapy. Cell.

[49] Hangauer M.J., Viswanathan V.S., Ryan M.J., Bole D., Eaton J.K., Matov A., Galeas J., Dhruv H.D., Berens M.E., Schreiber S.L. (2017). Drug-tolerant persister cancer cells are vulnerable to GPX4 inhibition. Nature.

[50] Liu Y., Peng B., Chen Z., Shen Y., Zhang J., Yuan X. (2024). Pan-cancer transcriptional atlas of minimal residual disease links DUSP1 to chemotherapy persistence. Exp. Hematol. Oncol..

[51] Ying H., Kimmelman A.C., Lyssiotis C.A., Hua S., Chu G.C., Fletcher-Sananikone E., Locasale J.W., Son J., Zhang H., Coloff J.L. (2012). Oncogenic Kras Maintains Pancreatic Tumors through Regulation of Anabolic Glucose Metabolism. Cell.

[52] Ling J., Kang Y., Zhao R., Xia Q., Lee D.-F., Chang Z., Li J., Peng B., Fleming J.B., Wang H. (2012). KrasG12D-Induced IKK2/β/NF-κB Activation by IL-1α and p62 Feedforward Loops Is Required for Development of Pancreatic Ductal Adenocarcinoma. Cancer Cell.

[53] Siveke J.T., Einwächter H., Sipos B., Lubeseder-Martellato C., Klöppel G., Schmid R.M. (2007). Concomitant Pancreatic Activation of KrasG12D and Tgfa Results in Cystic Papillary Neoplasms Reminiscent of Human IPMN. Cancer Cell.

[54] Crnogorac-Jurcevic T., Chelala C., Barry S., Harada T., Bhakta V., Lattimore S., Jurcevic S., Bronner M., Lemoine N.R., Brentnall T.A. (2013). Molecular Analysis of Precursor Lesions in Familial Pancreatic Cancer. PLoS One.

[55] Ascierto M.L., Worschech A., Yu Z., Adams S., Reinboth J., Chen N.G., Pos Z., Roychoudhuri R., Di Pasquale G., Bedognetti D. (2011). Permissivity of the NCI-60 cancer cell lines to oncolytic Vaccinia Virus GLV-1h68. BMC Cancer.

[56] Gallardo F., Schmitt D., Brandely R., Brua C., Silvestre N., Findeli A., Foloppe J., Top S., Kappler-Gratias S., Quentin-Froignant C. (2020). Fluorescent Tagged Vaccinia Virus Genome Allows Rapid and Efficient Measurement of Oncolytic Potential and Discovery of Oncolytic Modulators. Biomedicines.

[57] Marquette C.A., Petiot E., Spindler A., Ebel C., Nzepa M., Moreau B., Erbs P., Balloul J.-M., Quemeneur E., Zaupa C. (2024). 3D bioprinted CRC model brings to light the replication necessity of an oncolytic vaccinia virus encoding FCU1 gene to exert an efficient anti-tumoral activity. Front. Oncol..

[58] Carter M.E., Hartkopf A.D., Wagner A., Volmer L.L., Brucker S.Y., Berchtold S., Lauer U.M., Koch A. (2022). A Three-Dimensional Organoid Model of Primary Breast Cancer to Investigate the Effects of Oncolytic Virotherapy. Front. Mol. Biosci..

[59] Ahn B.Y., Jones E.V., Moss B. (1990). Identification of the vaccinia virus gene encoding an 18-kilodalton subunit of RNA polymerase and demonstration of a 5’ poly(A) leader on its early transcript. J. Virol..

[60] Heljasvaara R., Rodriguez D., Risco C., Carrascosa J.L., Esteban M., Rodriguez J.R. (2001). The Major Core Protein P4a (A10L Gene) of Vaccinia Virus Is Essential for Correct Assembly of Viral DNA into the Nucleoprotein Complex To Form Immature Viral Particles. J. Virol..

[61] Assarsson E., Greenbaum J.A., Sundström M., Schaffer L., Hammond J.A., Pasquetto V., Oseroff C., Hendrickson R.C., Lefkowitz E.J., Tscharke D.C. (2008). Kinetic analysis of a complete poxvirus transcriptome reveals an immediate-early class of genes. Proc. Natl. Acad. Sci..

[62] Rojo De La Vega M., Chapman E., Zhang D.D. (2018). NRF2 and the Hallmarks of Cancer. Cancer Cell.

[63] Wang R., An J., Ji F., Jiao H., Sun H., Zhou D. (2008). Hypermethylation of the Keap1 gene in human lung cancer cell lines and lung cancer tissues. Biochem. Biophys. Res. Commun..

[64] Singh A., Misra V., Thimmulappa R.K., Lee H., Ames S., Hoque M.O., Herman J.G., Baylin S.B., Sidransky D., Gabrielson E. (2006). Dysfunctional KEAP1–NRF2 Interaction in Non-Small-Cell Lung Cancer. PLoS Med..

[65] Namani A., Li Y., Wang X.J., Tang X. (2014). Modulation of NRF2 signaling pathway by nuclear receptors: Implications for cancer. Biochim. Biophys. Acta Mol. Cell Res..

[66] Fox D.B., Garcia N.M.G., McKinney B.J., Lupo R., Noteware L.C., Newcomb R., Liu J., Locasale J.W., Hirschey M.D., Alvarez J.V. (2020). NRF2 activation promotes the recurrence of dormant tumour cells through regulation of redox and nucleotide metabolism. Nat. Metab..

[67] França G.S., Baron M., King B.R., Bossowski J.P., Bjornberg A., Pour M., Rao A., Patel A.S., Misirlioglu S., Barkley D. (2024). Cellular adaptation to cancer therapy along a resistance continuum. Nature.

[68] Harding J.J., Telli M., Munster P., Voss M.H., Infante J.R., DeMichele A., Dunphy M., Le M.H., Molineaux C., Orford K. (2021). A Phase I Dose-Escalation and Expansion Study of Telaglenastat in Patients with Advanced or Metastatic Solid Tumors. Clin. Cancer Res..

[69] Riess J.W., Frankel P., Shackelford D., Dunphy M., Badawi R.D., Nardo L., Cherry S.R., Lanza I., Reid J., Gonsalves W.I. (2021). Phase 1 Trial of MLN0128 (Sapanisertib) and CB-839 HCl (Telaglenastat) in Patients With Advanced NSCLC (NCI 10327): Rationale and Study Design. Clin. Lung Cancer.

[70] Chen Y., Jiang Z., Li X. (2024). New insights into crosstalk between Nrf2 pathway and ferroptosis in lung disease. Cell Death Dis..

[71] Karki P., Angardi V., Mier J.C., Orman M.A. (2022). A Transient Metabolic State in Melanoma Persister Cells Mediated by Chemotherapeutic Treatments. Front. Mol. Biosci..

[72] Lin H., Wang L., Chen H., Shen Y., Wang C., Xue Y., Zheng Z., Zhang Y., Xia D., Wu Y. (2026). Mitochondrial fatty acid oxidation as the target for blocking therapy-resistance and inhibiting tumor recurrence: The proof-of-principle model demonstrated for ovarian cancer cells. J. Adv. Res..

[73] Shen S., Faouzi S., Souquere S., Roy S., Routier E., Libenciuc C., André F., Pierron G., Scoazec J.-Y., Robert C. (2020). Melanoma Persister Cells Are Tolerant to BRAF/MEK Inhibitors via ACOX1-Mediated Fatty Acid Oxidation. Cell Rep..

[74] Mancini C., Lori G., Pranzini E., Taddei M.L. (2024). Metabolic challengers selecting tumor-persistent cells. Trends Endocrinol. Metabol..

[75] Wicks E.E., Semenza G.L. (2022). Hypoxia-inducible factors: cancer progression and clinical translation. J. Clin. Investig..

[76] Ma Q., Zhang W., Wu K., Shi L. (2025). The roles of KRAS in cancer metabolism, tumor microenvironment and clinical therapy. Mol. Cancer.

[77] Russo M., Crisafulli G., Sogari A., Reilly N.M., Arena S., Lamba S., Bartolini A., Amodio V., Magrì A., Novara L. (2019). Adaptive mutability of colorectal cancers in response to targeted therapies. Science.

[78] Isozaki H., Sakhtemani R., Abbasi A., Nikpour N., Stanzione M., Oh S., Langenbucher A., Monroe S., Su W., Cabanos H.F. (2023). Therapy-induced APOBEC3A drives evolution of persistent cancer cells. Nature.

[79] Smith G.L., Benfield C.T.O., Maluquer De Motes C., Mazzon M., Ember S.W.J., Ferguson B.J., Sumner R.P. (2013). Vaccinia virus immune evasion: mechanisms, virulence and immunogenicity. J. Gen. Virol..

[80] Gong K., Guo G., Panchani N., Bender M.E., Gerber D.E., Minna J.D., Fattah F., Gao B., Peyton M., Kernstine K. (2020). EGFR inhibition triggers an adaptive response by co-opting antiviral signaling pathways in lung cancer. Nat. Cancer.

[81] Gong K., Guo G., Gerber D.E., Gao B., Peyton M., Huang C., Minna J.D., Hatanpaa K.J., Kernstine K., Cai L. (2018). TNF-driven adaptive response mediates resistance to EGFR inhibition in lung cancer. J. Clin. Investig..

[82] Schmitt M., Ceteci F., Gupta J., Pesic M., Böttger T.W., Nicolas A.M., Kennel K.B., Engel E., Schewe M., Callak Kirisözü A. (2022). Colon tumour cell death causes mTOR dependence by paracrine P2X4 stimulation. Nature.

[83] Falletta P., Sanchez-del-Campo L., Chauhan J., Effern M., Kenyon A., Kershaw C.J., Siddaway R., Lisle R., Freter R., Daniels M.J. (2017). Translation reprogramming is an evolutionarily conserved driver of phenotypic plasticity and therapeutic resistance in melanoma. Genes Dev..

[84] Sehgal K., Portell A., Ivanova E.V., Lizotte P.H., Mahadevan N.R., Greene J.R., Vajdi A., Gurjao C., Teceno T., Taus L.J. (2021). Dynamic single-cell RNA sequencing identifies immunotherapy persister cells following PD-1 blockade. J. Clin. Investig..

[85] Samson A., West E.J., Carmichael J., Scott K.J., Turnbull S., Kuszlewicz B., Dave R.V., Peckham-Cooper A., Tidswell E., Kingston J. (2022). Neoadjuvant Intravenous Oncolytic Vaccinia Virus Therapy Promotes Anticancer Immunity in Patients. Cancer Immunol. Res..

[86] Ma J., Ramachandran M., Jin C., Quijano-Rubio C., Martikainen M., Yu D., Essand M. (2020). Characterization of virus-mediated immunogenic cancer cell death and the consequences for oncolytic virus-based immunotherapy of cancer. Cell Death Dis..

[87] Dong W., Luo Y., He D., Zhang M., Zeng J., Chen Y. (2024). Oncolytic virotherapy against lung cancer: key receptors and signaling pathways of viral entry. Front. Immunol..

[88] Wang X., Zhou Q., Zhang X., Hu H., Liu B., Wang Y. (2025). Oncolytic viruses: a promising therapy for malignant pleural effusion and solid tumors. Front. Immunol..

[89] Béguin J., Foloppe J., Maurey C., Laloy E., Hortelano J., Nourtier V., Pichon C., Cochin S., Cordier P., Huet H. (2020). Preclinical Evaluation of the Oncolytic Vaccinia Virus TG6002 by Translational Research on Canine Breast Cancer. Mol. Ther. Oncolytics.

[90] Korsunsky I., Millard N., Fan J., Slowikowski K., Zhang F., Wei K., Baglaenko Y., Brenner M., Loh P.r., Raychaudhuri S. (2019). Fast, sensitive and accurate integration of single-cell data with Harmony. Nat. Methods.

[91] Saelens W., Cannoodt R., Todorov H., Saeys Y. (2019). A comparison of single-cell trajectory inference methods. Nat. Biotechnol..

[92] Street K., Risso D., Fletcher R.B., Das D., Ngai J., Yosef N., Purdom E., Dudoit S. (2018). Slingshot: cell lineage and pseudotime inference for single-cell transcriptomics. BMC Genom..

[93] Herreros-Pomares A., de-Maya-Girones J.D., Calabuig-Fariñas S., Lucas R., Martínez A., Pardo-Sánchez J.M., Alonso S., Blasco A., Guijarro R., Martorell M. (2019). Lung tumorspheres reveal cancer stem cell-like properties and a score with prognostic impact in resected non-small-cell lung cancer. Cell Death Dis..

