# Supplementary material for: Dissecting residual disease in spheroids reveals pan-cancer persistence signatures and a therapeutic window for oncolytic viruses
Source: Mol Ther Oncol. 2026 Jun 19;34(3):201279. doi: 10.1016/j.omton.2026.201279 (PMC13356641; doi:10.1016/j.omton.2026.201279)
Supplement: Document S1. Figures S1–S5 [file mmc1.pdf]

OMTON, Volume *34*

Supplemental information

Dissecting residual disease in spheroids reveals pan-cancer persistence signatures and a therapeutic window for oncolytic viruses

Clara Fauveau, Emily Lawendy, Jules Deforges, Sandrine Cochin, Baptiste Moreau, Jean-Marc Balloul, Philippe Erbs, Shreyansh Jain, Gilles Laverny, and PERSIST-SEQ Consortium

Table S1. Cluster markers. Genes significantly deregulated in each cluster compared to all the other clusters (genes with p-value < 0.05, log2 fold change > 0.25, expressed in at least 30% of cells in one group). See excel file *Tables S1-S8*.

Table S2. Gene importance scores derived from the trajectory analysis. The scoring indicates the relative contribution of a gene to the trajectory progression. See excel file *Tables S1-S8*.

Table S3. Deregulated genes upon treatment. Genes were selected if expressed in at least 30% of cells in one group. See excel file *Tables S1-S8*.

Table S4. Persistence markers. Genes significantly deregulated in the treated condition compared to the control (genes with p-value < 0.05, log2 fold change > 0.25, expressed in at least 30% of cells in one group). See excel file *Tables S1-S8*.

Table S5. Genes identified during the literature datamining. The persistence/resistance lists were compiled via PubMed queries using combinations of a gene name and resistance/persistence keywords mentioned in the abstract or title. See excel file *Tables S1-S8*.

Table S6. Core DTP Signatures. Genes consistently deregulated across preclinical DTP datasets (53 up- and 126 down-regulated, Figure 3C). See excel file *Tables S1-S8*.

Table S7. RT-qPCR primers and probes sequences. See excel file *Tables S1-S8*.

Table S8. Signatures used in GSEA analysis. See excel file *Tables S1-S8*.

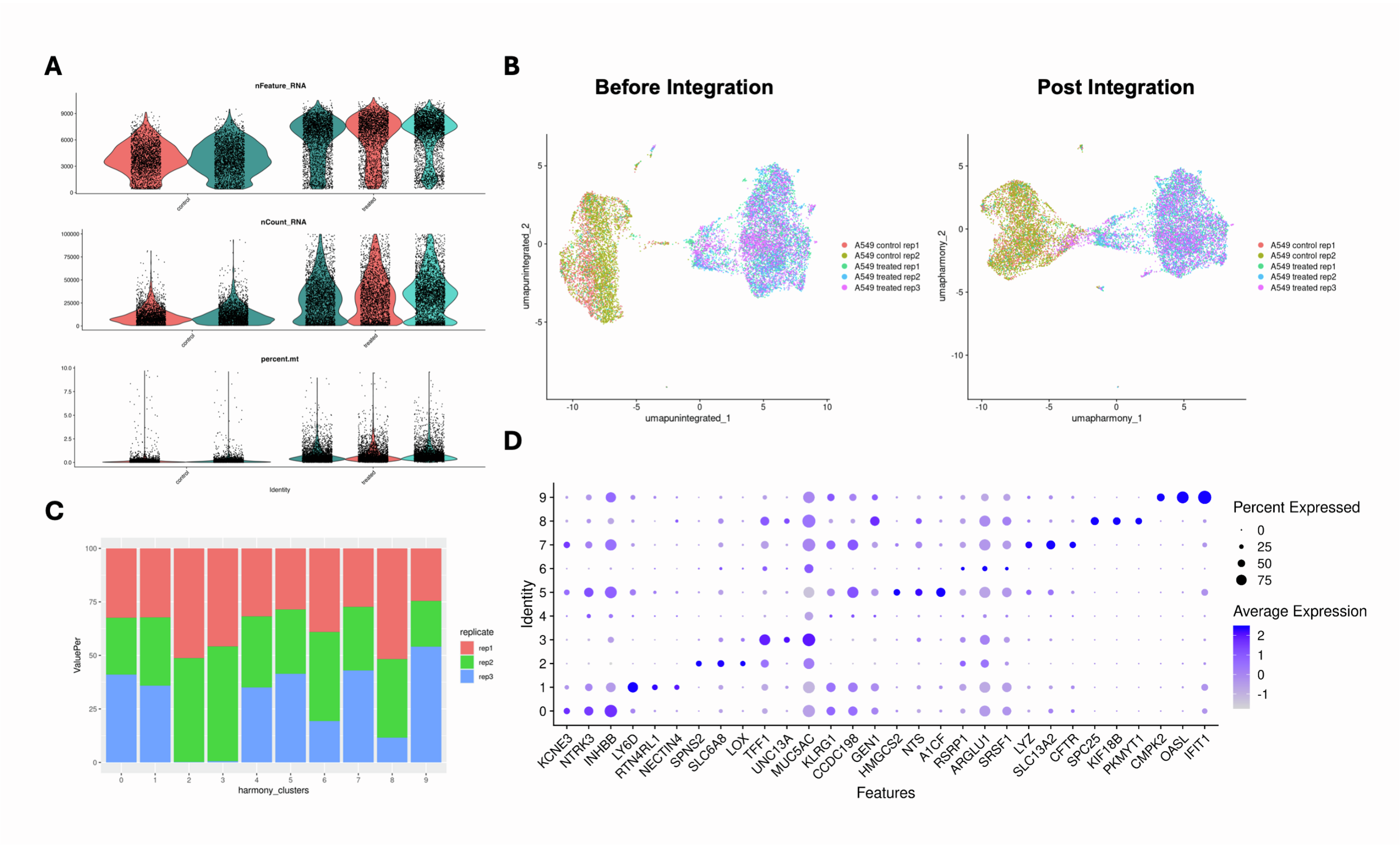

Figure S1. Single cell RNA-seq dataset analysis

(A) Violin plots of the number of unique genes detected in one cell (nFeatures), the number of reads per cell (nCount) and the percentage of mitochondrial genes (percent.mt) across samples.

(B) UMAP representation of the datasets before and after harmony integration.

(C) Replicates distribution per clusters.

(D) Dotplot representing the top three marker genes for each cluster.

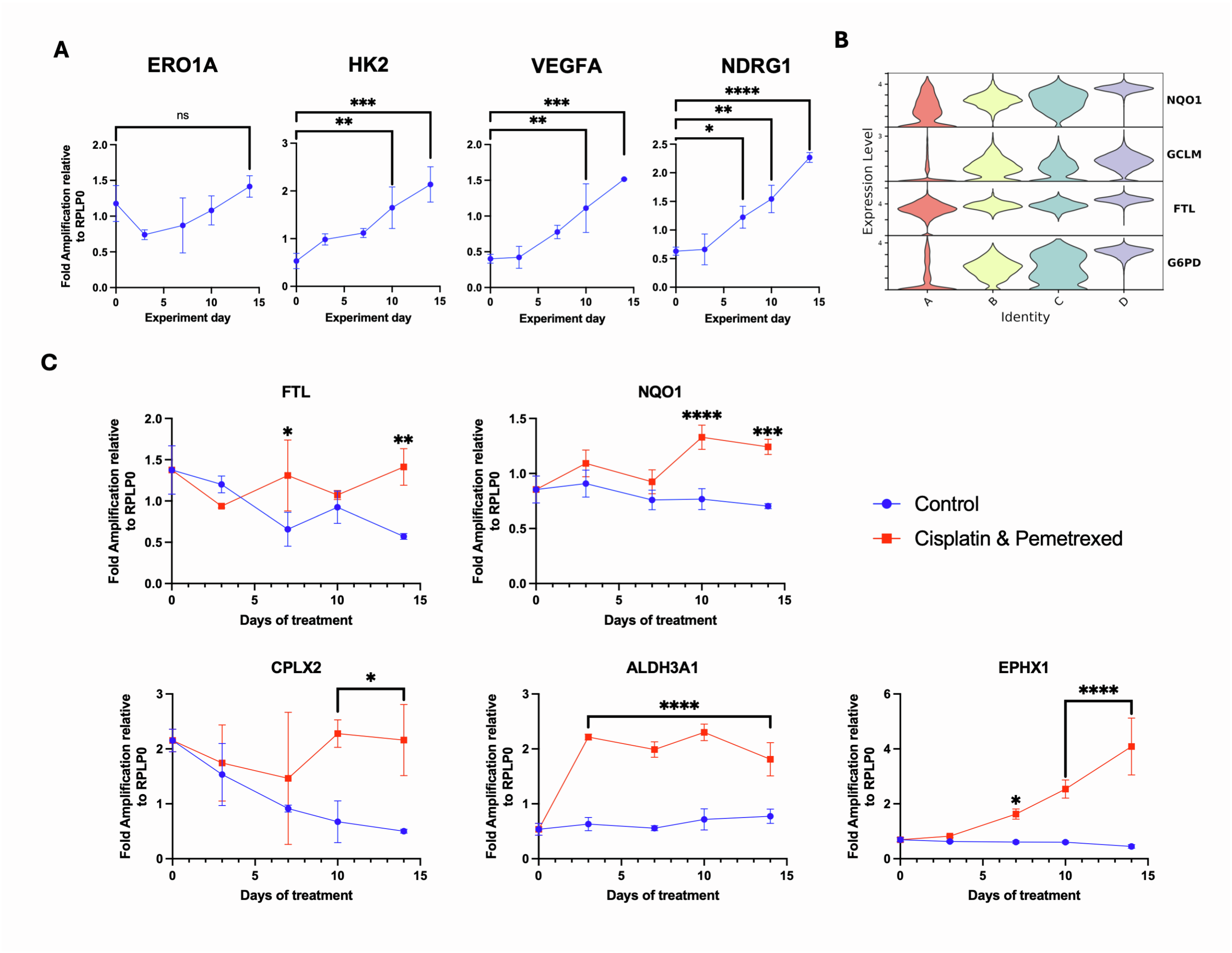

Figure S2. Hypoxia and NRF2 signature along the treatment and the trajectory

(A) Transcript levels of hypoxia-related genes in control A549 spheroids, determined by RT-qPCR, in three biological replicates. Mean (SD); ns $p \geq 0.05$, *$p < 0.05$, **$p < 0.01$, ***$p < 0.001$ and ****$p < 0.0001$ vs D0, determined using one-way ANOVA followed by a Dunnett's multiple comparisons test.

(B) Violin plots representing the transcript levels of deregulated NRF2 target genes across trajectory milestones.

(C) Transcript levels of NRF2 target genes in A549 spheroids treated with 10 µM cisplatin and 250 µM pemetrexed, or with vehicle for the indicated time, determined by RT-qPCR, in three biological replicates. Mean (SD); ns $p \geq 0.05$, *$p < 0.05$, **$p < 0.01$, ***$p < 0.001$ and ****$p <$

0.0001 vs matched control, determined using two-way ANOVA followed by a Šídák's multiple comparisons test.

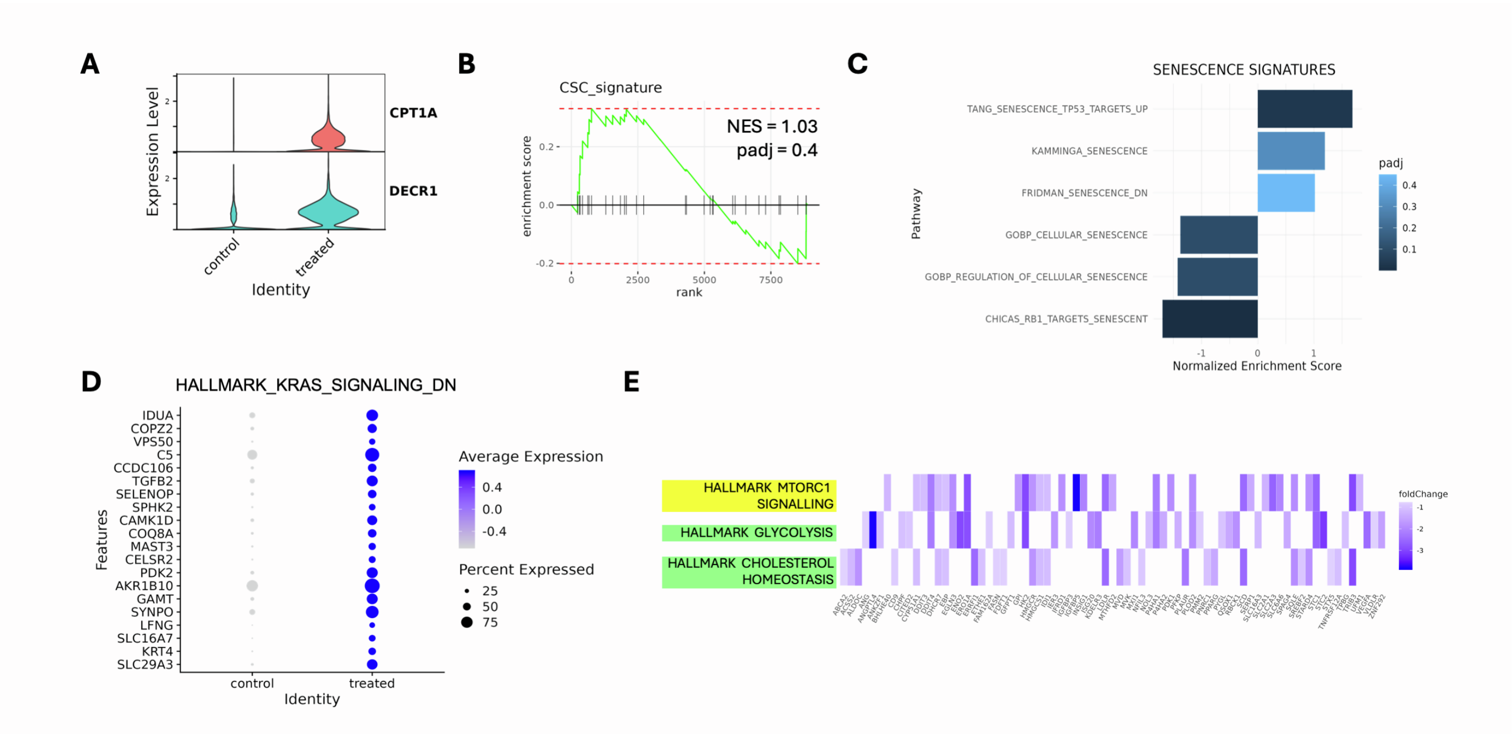

Figure S3. A549 spheroid signatures upon persistence

(A) Violin plots of significantly upregulated transcripts associated with fatty acid ß-oxidation under treatment.

(B) Enrichment plot of cancer stem cell signature[1] in persister cells. Adjusted p-value vs control cells computed using an adaptive multi-level split Monte-Carlo method.

(C) Enrichment scores of several senescent signatures in persistent spheroids. Adjusted p-value vs control cells computed using an adaptive multi-level split Monte-Carlo method.

(D) Dotplot of the top 20 genes deregulated upon treatment within the Hallmark KRAS signalling down gene set. colour scale represents the average expression, and dot size indicates the percentage of cells expressing each gene.

(E) Heatmap of the main deregulated genes driving the GSEA enrichment in the indicated hallmarks.

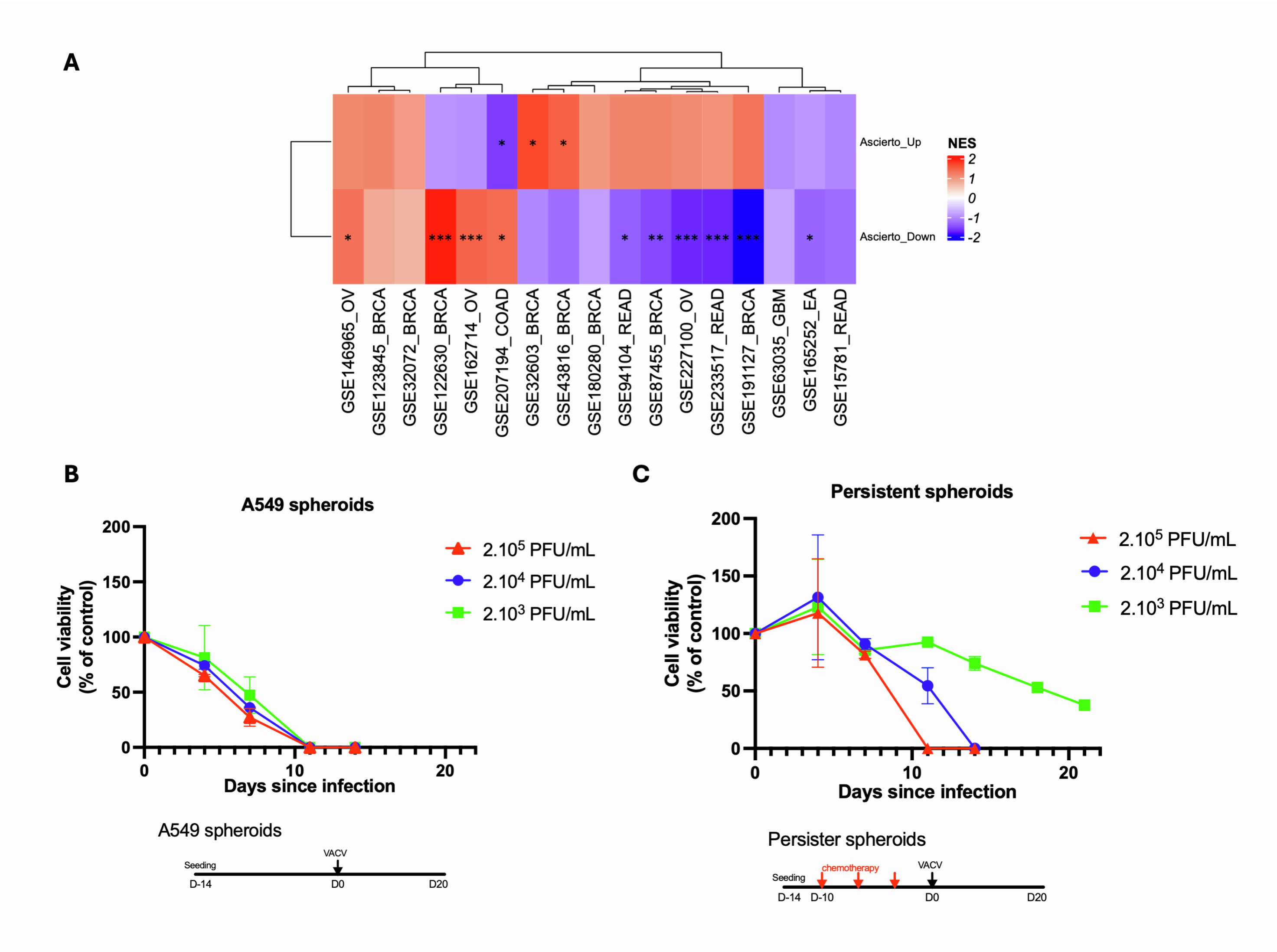

Figure S4. VACV in the context of persistence.

(A) Enrichment in signatures associated with permissivity to VACV oncolytic viruses[2] in MRD bulk datasets. The *Ascierto_UP* and *Ascierto_DOWN* signatures correspond to genes up- and down-regulated in cells that are more permissive to VACV infection, respectively. The colour scale represents the normalized enrichment score (NES) and the stars indicate the significance of the adjusted p-value. *$p < 0.05$, **$p < 0.01$ and ***$p < 0.001$ vs treatment-naïve samples, determined using an adaptive multi-level split Monte-Carlo method.

Cell viability of A549 spheroid (B) and persistent spheroids (C) treated with the indicated VACV concentration, expressed as a percentage of the vehicle. Total of six spheroids per condition/time point, in two biological replicates; mean (SD).

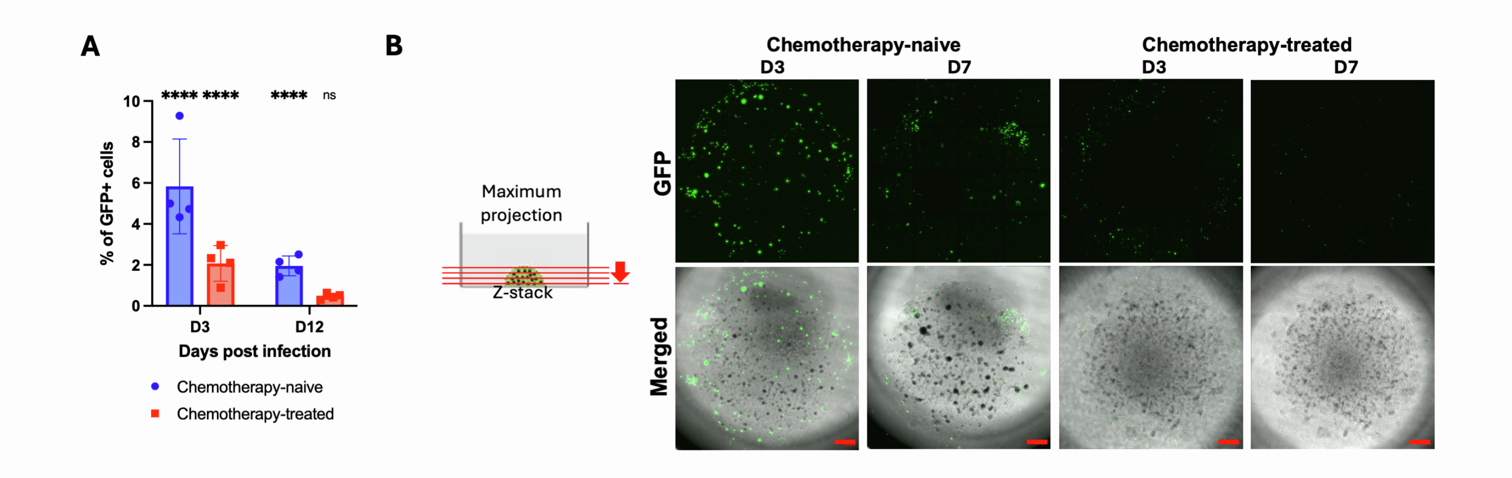

Figure S5. Virotherapy efficacy in naïve and chemotherapy-treated PDOs

(A) Percentage of GFP-positive cells three and twelve days post-infection in chemotherapy-naïve and chemotherapy-treated PDOs. Each dot represents one patient; mean (SD); ****p < 0.0001 vs matched uninfected PDOs, determined using two-way ANOVA followed by a Šídák's multiple comparisons test.

(B) Representative maximum intensity projections of GFP-expressing VACV merged with brightfield images of PDO hydrogel domes derived from patient #17, infected for the indicated time points. Scale bar: 1 mm.

References:

1. Herreros-Pomares, A., de-Maya-Girones, J.D., Calabuig-Fariñas, S., Lucas, R., Martínez, A., Pardo-Sánchez, J.M., Alonso, S., Blasco, A., Guijarro, R., Martorell, M., et al. (2019). Lung tumorspheres reveal cancer stem cell-like properties and a score with prognostic impact in resected non-small-cell lung cancer. Cell Death Dis *10*, 660. https://doi.org/10.1038/s41419-019-1898-1.

2. Ascierto, M.L., Worschech, A., Yu, Z., Adams, S., Reinboth, J., Chen, N.G., Pos, Z., Roychoudhuri, R., Di Pasquale, G., Bedognetti, D., et al. (2011). Permissivity of the NCI-60 cancer cell lines to oncolytic Vaccinia Virus GLV-1h68. BMC Cancer *11*, 451. https://doi.org/10.1186/1471-2407-11-451.
